# Noise robustness and metabolic load determine the principles of central dogma regulation

**Published:** 2024-01-08

**Authors:** Teresa W. Lo, Han Kyou James Choi, Dean Huang, Paul A. Wiggins

**Affiliations:** 1 Department of Physics, University of Washington, Seattle, Washington 98195, USA; 2 Department of Bioengineering, University of Washington, Seattle, Washington 98195, USA; 3 Department of Microbiology, University of Washington, Seattle, Washington 98195, USA

## Abstract

The processes of gene expression are inherently stochastic, even for essential genes required for growth. How does the cell maximize fitness in light of noise? To answer this question, we build a mathematical model to explore the trade-off between metabolic load and growth robustness. The model predicts novel principles of central dogma regulation: Optimal protein expression levels are vastly overabundant. Essential genes are transcribed above a lower limit of one message per cell cycle. Gene expression is achieved by load balancing between transcription and translation. We show that each of these novel regulatory principles is observed. These results reveal that robustness and metabolic load determine the global regulatory principles that govern central dogma processes, and these principles have broad implications for cellular function.

What rationale determines the optimal transcription and translation level of a gene in the cell? Protein expression levels optimize cell fitness [1, 2]: Too low of an expression level of essential proteins slows growth by compromising the function of essential processes [3], whereas the overexpression of proteins slows growth by increasing the metabolic load [4]. This trade-off naïvely predicts that the cell maximizes its fitness by a Goldilocks principle in which cells express just enough protein for function; however, achieving growth robustness is nontrivial, since all processes at the cellular scale are stochastic, including gene expression [5]. This biological noise leads to significant cell-to-cell variation in protein numbers, even for essential proteins that are required for growth [6, 7]. The optimal expression program must therefore ensure robust expression of hundreds of distinct essential gene products. In this paper, we explore the consequences of growth robustness on the central dogma regulatory program.

## Defining the RLTO Model.

To study the consequences of growth robustness on the central dogma quantitatively, we propose and analyze a minimal model: the Robustness-Load Trade-Off (RLTO) Model. The model includes three critical components: (i) Protein levels are stochastic and the single-cell growth rate depends upon them, (ii) gene transcription and translation generate a metabolic load, and (iii) cell growth is dependent on a large number of essential genes (*i.e*. high multiplicity). Implementing this model required a key theoretical innovation: the analysis of the consequences of noise on a highly-asymmetric fitness landscape. Our novel approach predicts new phenomenology absent from previous models (*e.g*. [8]).

**FIG. 1.**
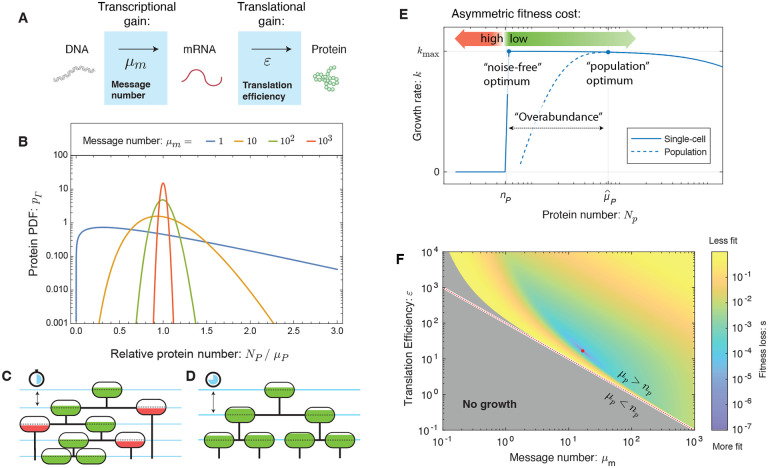
**Panel A: Two-stage amplifier model of expression.** The central dogma describes a two-step stochastic process by which genes are first transcribed and then translated. The transcription process transcribes an average of *μ*_*m*_ messages per cell cycle. Translation process translates an average of *ε* proteins per message. **Panel B: Gene-expression noise.** Due to the stochasticity of the central dogma processes, the protein number *N*_*p*_ is gamma-distributed. For high-expression genes, the protein number is tightly distributed about its mean; however, for low-expression genes, the distribution is extremely wide. **The RLTO Model.** A schematic cell lineage tree is shown during exponential growth. The cell fill represents the protein *i* number *N*_*p*_ relative to its threshold number *n*_*p*_ required for cell growth. **Panel C:** Reducing the mean expression level reduces the metabolic load (the spacing between blue lines); however, below-threshold cells (red fill) grow slowly. **Panel D:** Increasing protein expression increases the metabolic load (the spacing between blue lines); however, all cells are above threshold (green fill). **Panel E: The fitness landscape asymmetry drives protein overabundance.** The growth rate (blue line) for a single cell is zero for protein number *N*_*p*_ less than the threshold number *n*_*p*_ and decreases slowly for larger protein numbers as a consequence of the metabolic load. The population growth rate (dashed blue line) is a function of mean protein level *μ*_*p*_ and incorporates the stochasticity of *N*_*p*_. The growth rate is maximized at mean protein number ${\hat{\mu }}_{p}\gg n_{p}$, as a consequence of the fitness landscape asymmetry. **Panel F: Fitness landscape determines optimal message number and translation efficiency.** The fitness loss (*s ≡* ln *k*_max_*/k*) is shown as a function of message number (*μ*_*m*_) and translation efficiency (*ε*). The red dotted curve represents programs where the mean protein number is equal to the threshold (*μ*_*p*_ = *n*_*p*_) and the red dot represents the optimal regulatory program $\left({\hat{\mu }}_{m},\;\hat{\varepsilon }\right)$.

The protein number *N*_*p*_ expressed from gene *i* can be understood as the product of the gains of a two-stage amplification process: transcription followed by translation. (See Fig. 1A.) We will assume the numbers of proteins for gene *i* are gamma-distributed independent random variables [7, 9, 10], where the distribution is described by two gene-specific statistical parameters: *message number* (*μ*_*m*_), defined as the mean number of messages transcribed per cell cycle for gene *i*, and the *translation efficiency* (*ε*), the mean number of proteins translated from each message transcribed for gene *i*. The mean protein abundance is their product: *μ*_*p*_ = *μ*_*m*_*ε*. The metabolic load of transcription and translation is [4]:

(1) $$\frac{k_{0}}{k}=1+\frac{\lambda +\varepsilon }{N_{0}}\mu_{m},$$

where *k*_0_ is the growth rate in the absence of the metabolic load of gene *i*, *N*_0_ is the total metabolic load of all genes (in protein equivalents) and *λ* is the metabolic message cost. (See Supplementary Material Sec. A 1.) The *λ*-term represents the metabolic cost of transcription and the *ε*-term represents the metabolic cost of translation of gene *i*. We define the relative load as $\Lambda \equiv \lambda /N_{0}$ as the ratio of the metabolic load of a single message to the total metabolic cost of the cell. In *E. coli*, we estimate that Λ is roughly 10^*−*5^ and it is smaller still for eukaryotic cells. (See Supplementary Material Sec. A 17.)

The final task is to link protein expression with cellular function. Motivated by the concept of rate-limiting reactants [11] as well as single-cell growth dynamics of essential gene knockouts [12, 13], we will propose that the essential processes of the cell have a threshold-like dependence on each essential protein: We will assume that each essential protein *i* has a critical gene-specific threshold number *n*_*p*_ such that growth arrests below this critical number. (See Fig. 1CDE.) In our analysis, we will treat the thresholds *n*_*p*_ as gene-specific unknown parameters. The relative growth rate can be computed analytically:

(2) $$\text{ln}\frac{k}{k_{0}}=-\left(\Lambda +\frac{\varepsilon }{N_{0}}\right)\mu_{m}-\frac{1}{\text{ln}2}\gamma \left(\frac{\mu_{m}}{\text{ln}2},\frac{n_{p}}{\varepsilon \text{ln}2}\right),$$

where *γ* is the regularized incomplete gamma function, which is the CDF of the gamma distribution and represents the probability of arrest due to gene *i*. (See Supplementary Material Sec. A 1.) Eq. 2 represents an explicit analytic model for cell fitness that accounts for growth robustness to noise, metabolic load, and high multiplicity. In summary, the model depends only on a single global parameter: the relative metabolic load Λ and three gene-specific parameters: the threshold number *n*_*p*_, the message number *μ*_*m*_ and the relative translation efficiency *ε/N*_0_. We propose that the cell is regulated to maximize the growth rate with respect to transcription (message number) and translation (translation efficiency). The fitness landscape predicted by the RLTO model for representative parameters is shown in Fig. 1F.

## RLTO predicts protein overabundance.

The optimal regulatory program (*μ*_*m*_ and *ε* values) can be predicted analytically. They depend on only a single global parameter, the relative load Λ, and the gene-specific threshold number *n*_*p*_. Since the threshold number is not directly experimentally observable, we will predict the optimal overabundance *o*, defined as the ratio of the mean protein number to the threshold number:

(3) $$o\equiv \mu_{p}/n_{p}.$$

**FIG. 2.**
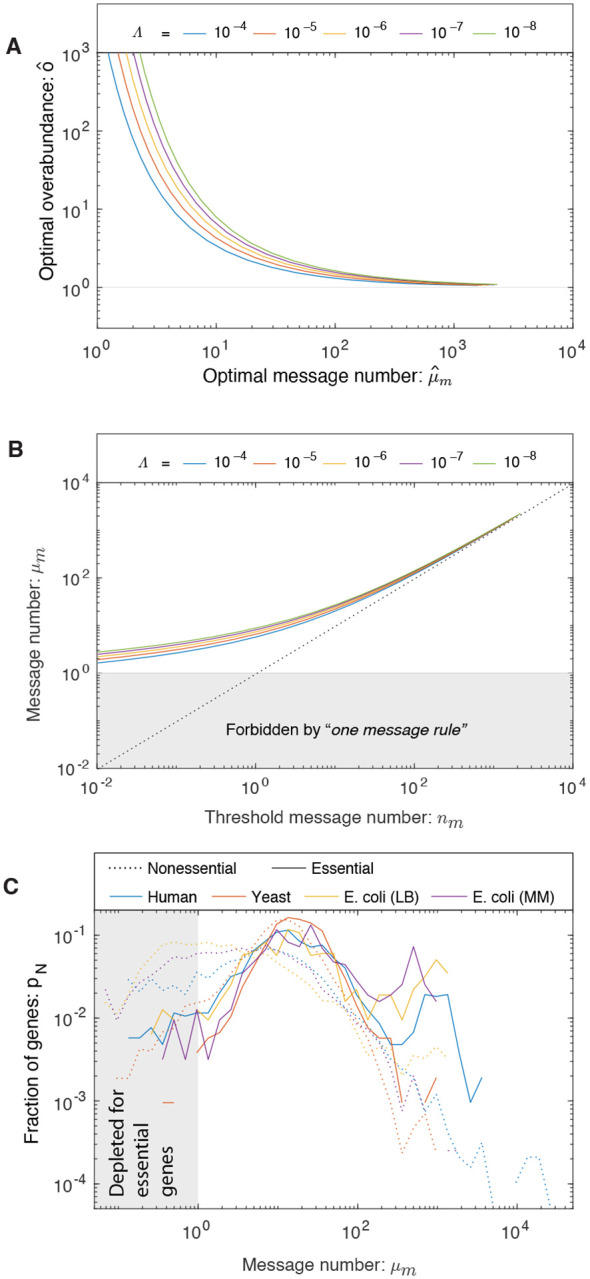
**Panel A: Overabundance is optimal for all genes.** For high-expression genes, low overabundance is optimal (*μ*_*p*_ *≈ n*_*p*_); however, for low-expression genes, vast overabundance is optimal (*μ*_*p*_ ≫ *n*_*p*_). From a quantitative perspective, overabundance depends on the relative load Λ; however, the qualitative dependence is invariant to over an orders-of-magnitude range of values. **Panel B: RLTO predicts the one-message-rule.** For high-expression genes, overabundance is low and the message number *μ*_*m*_ is predicted to be comparable to the threshold level *n*_*m*_ (dotted line); however, for low-expression genes there is a lower threshold (*μ*_*m*_ ≥ 1) below which expression is too noisy for robust growth. The threshold is weakly dependent on relative load Λ. **Panel C: A one message threshold is observed in three evolutionarily-divergent organisms.** As predicted by the RLTO model, essential, but not nonessential genes, are observed to be expressed above a one message per cell-cycle threshold. All organisms have roughly similar distributions of message number for essential genes, which are not observed for message numbers below a couple per cell cycle. This threshold is observed in *E. coli* under both rapid and slow growth conditions, as well as in yeast and human.

As shown in Fig. 2A, The RLTO model generically predicts that the optimal protein fraction is overabundant (*o* > 1); however, the overabundance is not uniform for all proteins. For highly-transcribed genes $\left(\mu_{m}\gg 1\right)$ like ribosomal genes, the overabundance is predicted to be quite small (*o* ≈ 1); however, for message numbers approaching unity, the overabundance is predicted to be extremely high (o≫1) At a quantitative level, the relation between optimal overabundance and message number depends on the relative load (Λ), but its phenomenology is qualitatively unchanged over orders of magnitude variation in Λ.

## Understanding the rationale for overabundance.

To explore both the robustness of the protein overabundance prediction and to understand its mathematical rationale, we explored a collection of more complex models numerically. (Supplementary Material Sec. B.) The key mathematical feature that drives overabundance is not the assumption of growth arrest, but rather the strong asymmetry of the fitness landscape: the high cost of protein *underabundance* and the low cost of protein *overabundance*. (See Fig. 1E.) In the RLTO model, this asymmetry is parameterized by the relative load (Λ), defined as the relative metabolic cost of transcribing an addition message. Since we estimate that Λ < 10^*−*5^, this cost is very low relative to the total metabolic cost of the cell, therefore we expect this asymmetry, and the prediction of the RLTO model, to be robust.

## Overabundance is observed in a range of experiments.

The RLTO Model predicts that all essential proteins are overabundant. In general, the RLTO model predicts that protein numbers have very significant robustness (*i.e*. buffering) to protein depletion. Although this result is potentially surprising, it is in fact consistent with many studies. For instance, Belliveau *et al*. have recently analyzed the abundance of a wide range of metabolic and other essential biological processes, and conclude that protein abundance appears to be in significant excess of what is required for function [14]. Likewise, CRISPRi approaches have facilitated the characterization of essential protein depletion. The qualitative results from these experiments are consistent with overabundance: Large-magnitude protein depletion is typically required to generate strong phenotypes [3, 15, 16]. In particular, Peters *et al*. engineered a complete collection of CRISPRi essential-gene depletion constructs in *Bacillus subtilis*. Importantly, when *dcas9* is constitutively expressed, these constructs deplete essential proteins about three-fold below their endogenous expression levels [3]; however, roughly 80% grew without measurable fitness loss in log-phase growth despite the depletion. When grouped by functional category, only ribosomal proteins were found to have statistically significant reductions in fitness [3]. As shown in Fig. 2B, the RLTO model predicts that all but the highest expression proteins are expected to show minimal fitness reductions in response to a three-fold depletion of essential enzymes. The optimality of protein overabundance explains the paradox of protein expression levels being simultaneously optimal [1] and in excess of what is required for function [3, 13, 14, 16]. Although this qualitative picture of essential protein overabundance is clear, there has yet to be a quantitative and detailed measurement of protein overabundance, and in particular, an analysis of the relationship between protein overabundance and message number.

## RLTO predicts a one-message transcription threshold.

The RLTO model predicts protein overabundance, but is there a clear transcriptional signature? To analyze this question, we define the message threshold $n_{m}\equiv \mu_{m}/o$. (This parameterization is convenient since it is independent of the translation efficiency.) We can then analyze the relation between optimal message number and threshold message number, as shown in Fig. 2B. The model predicts that even for genes that have extremely small threshold message numbers (*e.g. n*_*m*_ = 10^*−*2^), the optimal message number stays above one message transcribed per cell cycle. Qualitatively, expressing messages below this level is simply too noisy even for proteins needed at the lowest expression levels. The model therefore predicts a lower floor on transcription for essential genes of one message per cell cycle.

## A lower threshold is observed for message number.

To identify a putative transcriptional floor, we consider the central dogma in three model organisms: the bacterium *E. coli*, *Saccharomyces cerevisiae* (yeast) and *Homo sapiens* (human). For *E. coli*, we consider both rapid and slow growth conditions. We analyze three different transcriptional statistics for each gene: transcription rate, cellular message number, defined as the average number of messages instantaneously, and message number (*μ*_*m*_), defined as the number of messages transcribed in a cell cycle. Analysis of these organisms explores orders-of-magnitude differences in characteristics of the central dogma, including total message number, protein number, doubling time, message lifetime, and number of essential genes. (See Supplementary Tab. S2.) We hypothesize that cells must express essential genes above some threshold message number for robust growth; however, we expect to see that non-essential genes can be expressed at much lower levels since growth is not strictly dependent on their expression. To identify the putative transcriptional threshold, we generated histograms of each statistic in each organism and growth condition. As expected, there does not appear to be any consistent lower threshold between *E. coli*, yeast, or human transcription, either as characterized by the transcription rate or the cellular message number. (See Supplementary Fig. S10.) However, as predicted by the RLTO model, there is a consistent lower limit on message number (*μ*_*m*_) of roughly one message per cell cycle for essential genes. (See Fig. 2C.) This floor is consistent not only between *E. coli*, growing under two different conditions, but also between the three highly-divergent organisms: *E. coli*, yeast and human. We will conservatively define the minimum message number as: *μ*_*m*_ ≥ 1, and summarize this regulatory principle as the *one-message-rule* for essential gene expression.

## Translation efficiency is predicted to increase with transcription.

**FIG. 3.**
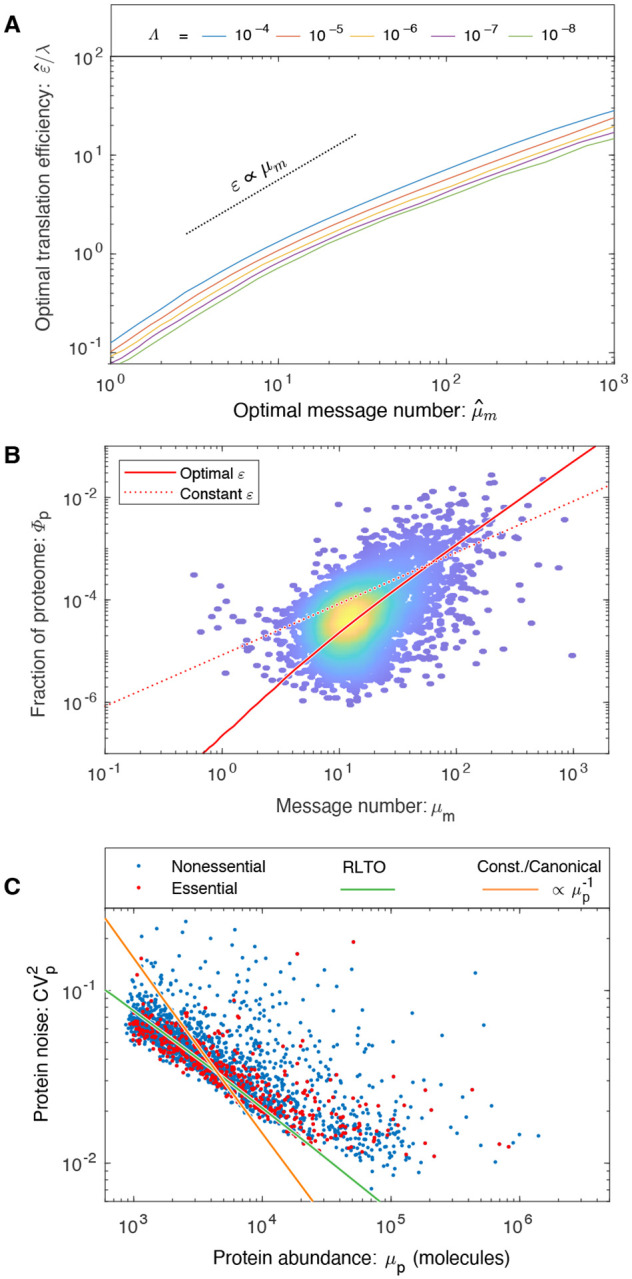
**Panel A: Load balancing.** The ratio of the optimal translation efficiency (*ε*ˆ) to the message cost (*λ*) is roughly independent of the relative load (Λ). The translation efficiency *ε* is predicted to be roughly proportional message number *μ*_*m*_. **Panel B: RLTO predicts the protein-message-abundance relation in yeast.** The observed proteome fraction is compared to two models: the RLTO optimal model (solid red line) and constant-translation-efficiency model (dotted red line). Both models make parameter-free predictions. The RLTO optimum predicts the global trend. (Data from Ref. [17].) **Panel C: RLTO predicts the magnitude of noise in Yeast.** The observed gene expression noise is yeast is shown for essential and non-essential genes. Two protein-message abundance models are compared to the data: The RLTO model (green) versus the constant-translation-efficiency (canonical model, orange). The RLTO model predicts both the magnitude of the noise, as well as its scaling with protein abundance. The reduced slope of the RLTO versus the canonical model prediction is the consequence of load balancing, which reduces the noise for the noisiest, low-expression genes. (Data from Ref. [6].)

How is the process of gene expression optimally balanced between transcription and translation? Maximization of the growth rate (Eq. 2) with respect to the translation efficiency can also be performed analytically, predicting the optimal translation efficiency, shown Fig. 3A. We provide an exact expression in the Supplementary Material Sec. A 3; however, an approximate expression for the translation efficiency is more clearly interpretable:

(4) $$\hat{\varepsilon }\approx 0.1\lambda {\hat{\mu }}_{m}.$$

The optimal translation efficiency has two important qualitative features for central dogma regulation. The first prediction is that as the message cost (*λ*) rises, the optimal translation efficiency $\left(\hat{\varepsilon }\right)$ increases in proportion while the message number decreases. We present evidence for this prediction in the Supplementary Material Sec. A 10.

The second prediction is that the optimal translation efficiency is also approximately proportional to message number $\left(\hat{\varepsilon }\propto \mu_{m}\right)$. Therefore, the RLTO model predicts that low expression levels should be achieved with low levels of transcription and translation, whereas high expression genes are achieved with high levels of both. We call this relation between optimal transcription and translation th *load balancing* principle. The most direct test of load balancing is measuring the protein-message abundance relation. Due to load balancing, the RLTO model predicts protein number (and proteome fraction) scale like:

(5) $${\hat{\mu }}_{p}\propto {\hat{\mu }}_{m}^{2},$$

whereas a constant-translation-efficiency model has linear scaling $\left(\mu_{p}\propto \mu_{m}\right)$. Computing proteome fraction, rather than protein number, results in a parameter-free prediction. (See Supplemental Sec. A 11.)

## RLTO predicts proteome fractions in eukaryotic cells.

To test the RLTO predictions, we compare observed proteome measurements in three evolutionarily divergent species, *E. coli* [18], yeast [17] and mammalian cells [19], to two models: the RLTO and the constant-translation-efficiency models. The results of the parameter-free predictions are shown in Fig. 3B for yeast and in Supplemental Fig. S7 for *E. coli* and mammalian cells, respectively. The RLTO model clearly captures the global trend in the proteome-fraction message-number relation in eukaryotic cells.

In *E. coli*, the constant-translation-efficiency model better describes the data. Why does this organism appear not to load balance? In the supplementary material, we demonstrate that the observed translation efficiency is consistent with the RLTO model, augmented by a ribosome-per-message limit. Hausser *et al*. have proposed just such a limit, based on the ribosome footprint on mRNA molecules [8]. (See Supplementary Material Sec. A 16.) Although this augmented model is consistent with central dogma regulation in *E. coli*, it is not a complete rationale. This proposed translation-rate limit could be circumvented by increasing the lifetime of *E. coli* messages, which would increase the translation efficiency. A more in-depth analysis specific to *E. coli* is needed to understand why the observed message lifetime is so short.

## RLTO model predicts observed noise in yeast.

Although the protein fraction measurements support the RLTO predictions for the translation efficiency in eukaryotic cells, these measurements do not provide a compelling rationale for why load balancing maximizes the growth rate. To understand its rationale, we explore its implications for noise. The Telegraph model predicts that the noise should be inversely to the message number [9, 10]:

(6) $${\text{CV}}_{p}^{2}=\frac{\text{ln}2}{\mu_{m}},$$

however, it is the relation between mean protein abundance *μ*_*p*_ and noise $\left({\text{CV}}_{p}^{2}\right)$ which is typically reported [6, 7]. The scaling of the optimal translation efficiency with the message number in eukaryotic cells (Eq. 4) predicts that noise should scale with protein abundance ${\text{CV}}_{p}^{2}\propto \mu_{p}^{-1/2}$ in yeast; however, due to the observed absence of translation-efficiency scaling in bacteria, the noise should scale as ${\text{CV}}_{p}^{2}\propto \mu_{p}^{-1}$ in bacteria, as observed [7]. Does the yeast noise show the predicted scaling? The parameter-free RLTO noise prediction closely matches the observed noise in both magnitude and scaling, as shown in Fig. 3C.

## Reducing noise is the rationale for load balancing.

This noise analysis also provides a conceptual insight into the rationale for load balancing. The load balanced (RLTO-green) and constant-translation-efficiency (orange) predictions for the noise are shown in Fig. 3C. Load balancing results in decreased noise for low-expression, noisy genes over what is achieved with constant translation efficiency. This decreased noise is predicted to increase growth robustness. In principle, the noise could be reduced further by tipping the balance even more towards transcription; however, the RLTO model predicts that this approach is too metabolically costly, and the optimal strategy is that observed for noise scaling in yeast.

## DISCUSSION

### Implications for non-essential genes.

In our analysis, we have focused on essential genes in order to motivate the growth-threshold in the RLTO model. To what extent do non-essential genes share the same optimization? In support of the proposal that RLTO optima describe non-essential genes is the success of the model in predicting the translation efficiency for all genes, not just essential genes. (See Fig. 3.) Furthermore, the definition of a gene as *essential* depends on context: For instance, in the context of *E. coli* growth on lactose, the gene *lacZ* is essential, although it is non-essential on other carbon sources [20]. Under growth conditions where the *lacZ* gene is essential, we predict that LacZ should be overabundant, consistent with observation [21]. Finally, our modeling suggested that RLTO model phenomenology is the results of asymmetry of the cost of under versus overabundance. For non-essential genes whose activity significantly increase fitness, we still expect fitness asymmetry due to the low relative metabolic cost of increased expression. We therefore expect all gene products, most especially those with low expression, to be overabundant, under conditions where their activity increases fitness.

### Implications of overabundance for inhibitors.

The generic nature of overabundance, especially for low-expression proteins, has important potential implications for the targeting of these proteins with small-molecule inhibitors (*e.g*. drugs). For the highest expression proteins, like the constituents of the ribosome, relatively small decreases in the active fraction (*e.g*. a three-fold reduction) are expected to lead to growth arrest [3]. This may help explain why inhibitors targeting translation make such effective antimicrobial drugs. (See Fig. 2B.) However, we predict that the lowest expression proteins require a much higher fraction of the protein to be inactivated, with the lowest-expression proteins expected to need more than a 100-fold depletion. This predicted robustness makes these proteins much less attractive drug targets [22].

### The principles that govern central dogma regulation.

**FIG. 4.**
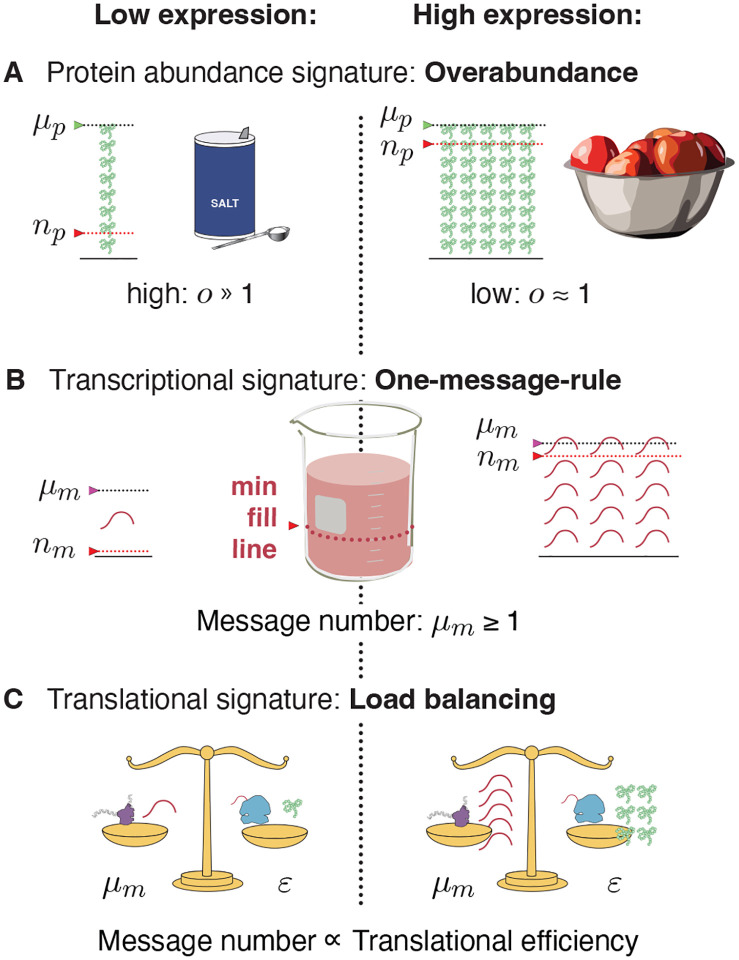
Central dogma regulatory principles. **Panel A: Overabundance.** Low-expression essential genes are expressed with high overabundance; whereas, high-expression essential genes are expressed with low overabundance. Pie-baking analogy: The low cost of essential low-quantity ingredients (*e.g*. salt) makes it optimal to buy them in great excess, while the higher cost of essential large-quantity ingredients (*e.g*. apples) makes it optimal to buy only what is needed. **Panel B: One-message-rule.** Robust expression of essential genes requires them to be transcribed above a threshold of one message per cell cycle. **Panel C: Load balancing.** In eukaryotic cells, optimal fitness is achieved by balancing transcription and translation: The optimal message number is proportional to the optimal translation efficiency. High (low) expression levels are achieved by high (low) levels of transcription followed by high (low) levels of translation per message.

What are the biological implications of noise? We propose that robustness to noise fundamentally shapes the central dogma regulatory program for all genes and predicts a number of key regulatory principles. (See Fig. 4.) For high-expression genes, load balancing implies that gene expression consists of both high-amplification translation and transcription. The resulting expression level has low overabundance relative to the threshold required for function. In contrast, for essential low-expression genes, a three-fold strategy is implemented: (i) overabundance raises the mean protein levels far above the threshold required for function, (ii) load balancing and (iii) the one message rule ensures that message number is sufficiently large to lower the noise of inherently-noisy low-expression genes. We anticipate that these regulatory principles, in particular protein overabundance, will have significant impact, not only on our understanding of central dogma regulation specifically, but in understanding the rationale for protein expression level and function in many biological processes.

## Supplementary Material

1

## Data Availability

We include a source data file which includes the estimated message numbers as well as essential/nonessential classifications for each organism.
