# Noise robustness and metabolic load determine the principles of central dogma regulation

**Published:** 2024-05-23

**Authors:** Teresa W. Lo, Han James Choi, Dean Huang, Paul A. Wiggins

**Affiliations:** 1 Department of Physics, University of Washington, Seattle, Washington 98195, USA; 2 Department of Bioengineering, University of Washington, Seattle, Washington 98195, USA; 3 Department of Microbiology, University of Washington, Seattle, Washington 98195, USA

## Abstract

The processes of gene expression are inherently stochastic, even for essential genes required for growth. How does the cell maximize fitness in light of noise? To answer this question, we build a mathematical model to explore the trade-off between metabolic load and growth robustness. The model predicts novel principles of central dogma regulation: Optimal protein expression levels for many genes are in vast overabundance. Essential genes are transcribed above a lower limit of one message per cell cycle. Gene expression is achieved by load balancing between transcription and translation. We present evidence that each of these novel regulatory principles is observed. These results reveal that robustness and metabolic load determine the global regulatory principles that govern central dogma processes, and these principles have broad implications for cellular function.

## One-sentence summary

Fitness maximization predicts protein overabundance, a transcriptional floor, and the balancing of transcription and translation.

## INTRODUCTION

What rationale determines the optimal transcription and translation level of a gene in the cell? Protein expression levels optimize cell fitness [1, 2]: Too low of an expression level of essential proteins slows growth by compromising the function of essential processes [3, 4], whereas the overexpression of proteins slows growth by increasing the metabolic load [5]. This trade-off naïvely predicts that the cell maximizes its fitness by a Goldilocks principle in which cells express just enough protein for function [6]; however, achieving growth robustness is nontrivial, since all processes at the cellular scale are stochastic, including gene expression [7]. This biological noise leads to significant cell-to-cell variation in protein numbers, even for essential proteins that are required for growth [8, 9]. The optimal expression program must therefore ensure robust expression of hundreds of distinct essential gene products. In this paper, we explore the consequences of growth robustness on the central dogma regulatory program.

## RESULTS

### Defining the RLTO Model.

To study the consequences of growth robustness on the central dogma quantitatively, we propose and analyze a minimal model: the Robustness-Load Trade-Off (RLTO) Model. The model includes three critical components: (i) Protein levels are stochastic and the single-cell growth rate depends upon them, (ii) gene transcription and translation generate a metabolic load, and (iii) cell growth is dependent on a large number of essential genes. These model characteristics result in a highly-asymmetric fitness landscape. The optimization of expression on this asymmetric landscape predicts new phenomenology absent from previous models (*e.g.* [10]).

**FIG. 1.**
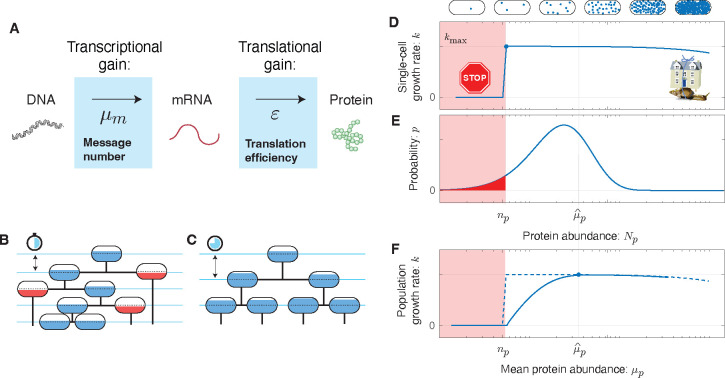
The RLTO Model. **Panel A: Gene expression is stochastic.** The central dogma describes a two-stage stochastic process in which genes are first transcribed and then translated. The transcription process transcribes an average of $\mu_{m}$ messages per cell cycle. The translation process translates an average of ε proteins per message. **Panel B & C:** A schematic cell lineage tree is shown during exponential growth. For a specific protein i, the cell fill represents the protein number $N_{p}$ relative to its threshold number *n*_*p*_ required for cell growth. **Panel B:** Reducing the mean expression level reduces doubling time; however, stochasticity in expression results in below-threshold cells (red fill) which grow slowly. **Panel C:** Increasing protein expression increases the doubling time; however, all cells are above threshold (blue fill). **Panel D: The fitness landscape as a function of protein number.** Growth arrests for protein number $N_{p}$ smaller than the threshold level $n_{p}$ (red) due to the failure of essential processes. High expression levels are penalized due to the metabolic cost of protein expression. This trade-off leads to a highly asymmetric fitness landscape: The relative metabolic cost of overabundance is small relative to the cost of growth arrest due to the large size of the total metabolic load $N_{0}$. **Panel E: The gene expression process is stochastic.** There is significant cell-to-cell variation in protein abundance ($N_{p}$) around the mean level ($\mu_{p}$). Even for mean expression levels significantly above the threshold level $n_{p}$, some cells fall below threshold (red). The distribution in protein number is modeled using a gamma distribution [9], parameterized by message number $\mu_{m}$ and translation efficiency ε. **Panel F: The robustness load trade-off determine the optimal expression level.** The population growth rate depends on the distribution of the protein number. The asymmetry of the fitness landscape drives the optimal expression level far above the threshold level due to the high fitness cost of low protein abundance.

The protein number $N_{p}$ expressed from gene i is the product of two sequential stochastic processes: transcription and translation [11], leading to cell-to-cell variation in protein number, which we will refer to as *noise*. In our analysis, we will model gene expression using the canonical equilibrium Paulsson model [12]. (See Fig. 1A.) In this model, the numbers of proteins $N_{p}$ for gene i are predicted to be gamma-distributed [13], in close agreement with observation [9]. The distribution is described by two gene-specific statistical parameters: *message number* $\left(\mu_{m}\right)$, defined as the mean number of messages transcribed per cell cycle for gene i, and the *translation efficiency* (ε), the mean number of proteins translated from each message transcribed for gene i. The mean protein abundance is their product: $\mu_{p}=\mu_{m}\varepsilon$. These parameters can be expressed in terms of ratios of the rates of the underlying central dogma processes, as described in Supplementary Material Sec. A 1.

How should the effect of essential protein expression on growth rate be modeled in the context of the RLTO model? Much recent work has focused on cellular resource allocation to functional sectors (*e.g.* [14]). In this approach, an optimization is performed by coherently modulating the abundance of all protein in a particular sector, leading to a trade-off between functional capacities of the cell. However, in the RLTO model, the optimization is fundamentally different: We consider the incoherent variation in the abundance of protein species i due to noise. For these incoherent changes, we generically expect proteins to exhibit rate-limited kinetics: Increases in the protein number $N_{p}$ above a threshold level $n_{p}$ has minimal effect on the rate since other chemical species (proteins, metabolites, *etc.*) are rate limiting [15]. However, if the protein number $N_{p}$ falls below the threshold $n_{p}$, then protein species i becomes rate limiting and leads to a significant slowdown of the growth rate. In the RLTO model, we coarse-grain the details of this growth slowdown as growth arrest. (See Fig. 1BCD.) There is already some precedent for the use of this type of threshold (*e.g.* [16]), but we will demonstrate that the detailed form of the fitness landscape is not important. (See Supplementary Material Sec. B.) Although sufficiently detailed knowledge of the relevant molecular and cellular biology could be used to predict the protein thresholds $n_{p}$, we will treat these as gene-specific unknown parameters.

**FIG. 2.**
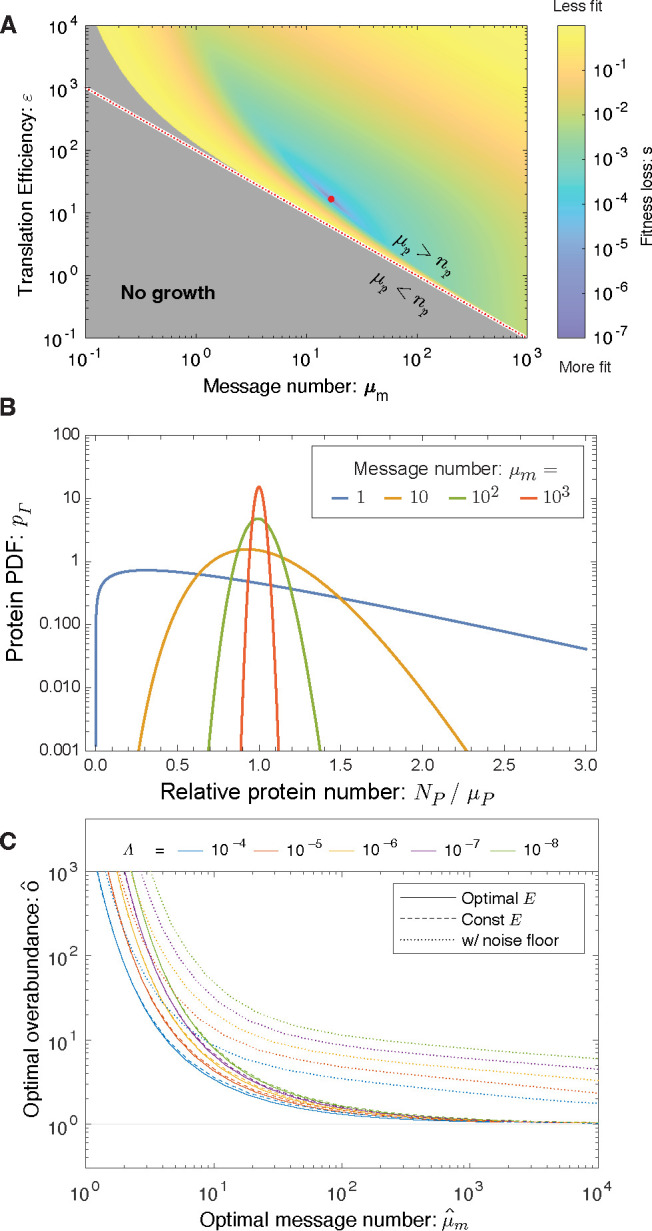
The RLTO model predicts overabundance is optimal. **Panel A: Fitness landscape determines optimal message number and translation efficiency.** The fitness loss $(s\equiv \left.lnk_{max}/k\right)$ is shown as a function of message number $\left(\mu_{m}\right)$ and translation efficiency (ε). The red dotted curve represents programs where the mean protein number is equal to the threshold ($\mu_{p}=n_{p}$) and the red dot represents the optimal regulatory program $\left({\overset{ˆ}{\mu }}_{m},\overset{ˆ}{\varepsilon }\right)$. **Panel B: Gene-expression noise.** Due to the stochasticity of the central dogma processes at equilibrium, the protein number $N_{p}$ is gamma-distributed [12]. For high-expression genes, expression has low noise and the protein number is tightly distributed about its mean; however, for low-expression genes, expression is noisy and the distribution is extremely wide. **Panel C: Overabundance is optimal for all genes.** For high-expression genes, low overabundance is optimal ($\mu_{p}\approx n_{p}$); however, for low-expression genes, vast overabundance is optimal $\left(\mu_{p}\gg n_{p}\right)$. From a quantitative perspective, overabundance depends on the relative load Λ; however, the qualitative dependence is invariant to over an orders-of-magnitude range of values.

As shown in Materials and Methods, the relative cellular fitness with respect to the expression of gene i can be computed by combining the fitness losses associated with robustness (Eq. 6) and metabolic load (Eq. 8):

(1) $$\frac{\Delta k}{k_{0}}=-(\Lambda +\frac{\varepsilon }{N_{0}})\mu_{m}-\frac{1}{\ln2}\gamma (\frac{\mu_{m}}{\ln2},\frac{n_{p}}{\varepsilon \ln2}),$$

where the first term represents fitness loss due to metabolic load of transcription and translation while the second term represents loss due to the arrest of essential processes. In summary, the model depends only on a single global parameter: the relative metabolic load Λ and three gene-specific parameters: the threshold number $n_{p}$, the message number $\mu_{m}$ and the relative translation efficiency $\varepsilon /N_{0}$. We propose that the cell is regulated to maximize the growth rate with respect to transcription (message number) and translation (translation efficiency). The fitness landscape predicted by the RLTO model for representative parameters is shown in Fig. 2A.

### RLTO predicts protein overabundance.

The optimal regulatory program ($\mu_{m}$ and ε values) can be predicted analytically. They depend on only a single global parameter, the relative load Λ, and the gene-specific threshold number $n_{p}$. Since the threshold number is not directly observable experimentally, we will instead predict the optimal overabundance o, defined as the ratio of the mean protein number to the threshold number:

(2) $$o\equiv \mu_{p}/n_{p}.$$

As shown in Fig. 2C, The RLTO model generically predicts that the optimal protein fraction is overabundant (o>1); however, the overabundance is not uniform for all proteins. For highly-transcribed genes $\left(\mu_{m}\gg 1\right)$ like ribosomal genes, the overabundance is predicted to be quite small (o≈1); however, for message numbers approaching unity, the overabundance is predicted to be extremely high (o≫1). At a quantitative level, the relation between optimal overabundance and message number depends on the relative load (Λ), but its phenomenology is qualitatively unchanged over orders of magnitude variation in Λ.

### Understanding the rationale for overabundance.

To explore both the robustness of the protein overabundance prediction and to understand its mathematical rationale, we explored a collection of more complex models numerically. (Supplementary Material Sec. B.) The key mathematical feature that drives overabundance is not the assumption of growth arrest, but rather the strong asymmetry of the fitness landscape: the high cost of protein *underabundance* and the low cost of protein *overabundance*. (See Fig. 1EF.) In the RLTO model, this asymmetry is parameterized by the relative load (Λ), defined as the relative metabolic cost of transcribing an additional message. Since we estimate that $\Lambda <10^{-5}$, this cost is very low relative to the total metabolic cost of the cell, therefore we expect this asymmetry, and the prediction of the RLTO model, to be robust.

### Overabundance is observed in a range of experiments.

The RLTO Model predicts that all essential proteins are overabundant. In general, the RLTO model predicts that protein numbers have very significant robustness (*i.e.* buffering) to protein depletion. Although this result is potentially surprising, it is in fact consistent with many studies. For instance, Belliveau *et al.* have recently analyzed the abundance of a wide range of metabolic and other essential biological processes, and conclude that protein abundance appears to be in significant excess of what is required for function [6]. Likewise, CRISPRi approaches have facilitated the characterization of essential protein depletion. The qualitative results from these experiments are consistent with overabundance: Large-magnitude protein depletion is typically required to generate strong phenotypes [3, 17, 18]. In particular, Peters *et al.* engineered a complete collection of CRISPRi essential-gene depletion constructs in *Bacillus subtilis*. Importantly, when *dcas9* is constitutively expressed, these constructs deplete essential proteins about three-fold below their endogenous expression levels [3]; however, roughly 80% grew without measurable fitness loss in log-phase growth despite the depletion. When grouped by functional category, only ribosomal proteins were found to have statistically significant reductions in fitness [3]. As shown in Fig. 2C, the RLTO model predicts that all but the highest expression proteins are expected to show minimal fitness reductions in response to a three-fold depletion of essential enzymes. The optimality of protein overabundance explains the paradox of protein expression levels being simultaneously optimal [1] and in excess of what is required for function [3, 4, 6, 18]. Although this qualitative picture of essential protein overabundance is clear, there has yet to be a quantitative and detailed measurement of protein overabundance, and in particular, an analysis of the relationship between protein overabundance and message number.

### RLTO predicts a one-message transcription threshold.

**FIG. 3.**
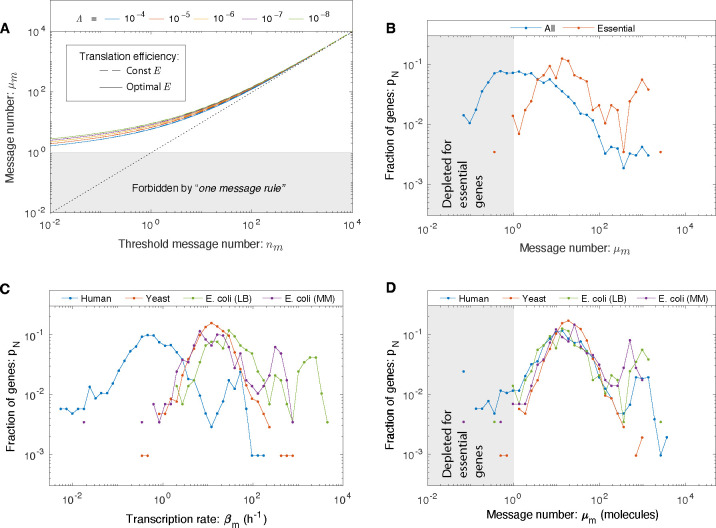
A lower threshold for transcription: The one message rule. **Panel A: RLTO predicts the one-message rule.** For high-expression genes, overabundance is low and the message number $\mu_{m}$ is predicted to be comparable to the threshold level *n*_*m*_ (dotted line); however, for low-expression genes there is a lower threshold ($\mu_{m}\geq 1$) below which expression is too noisy for robust growth. The threshold is weakly dependent on relative load Λ. **Panel B: A one-message threshold is observed in *E. coli* for essential genes.** A histogram shows the distribution of gene message numbers for all genes (blue) versus essential genes (orange). As predicted by the RLTO model, virtually all essential genes are expressed above the one-message-per-cell-cycle threshold. **Panel C: The distribution of transcription rates for essential genes.** No alignment is observed between the distributions of transcription rates in three evolutionarily-divergent organisms. For instance, the per gene transcription rate is significantly lower in human cells relative to *E. coli*. **Panel D: The distribution of message numbers for essential genes in three evolutionarily-divergent organisms.** The alignment of distributions of message number per gene between human, yeast, and *E. coli* (under two distinct growth conditions) reveals a nontrivial commonality between central dogma regulatory programs. We propose that the rationale for this alignment is the one-message rule that predicts that all essential genes must be expressed above one message per cell cycle. Both yeast and *E. coli* come very close to satisfying this proposed threshold; however, a greater proportion of genes in human break the one-message threshold. We speculate that this is due in part to the *ad hoc* nature of the essential-gene classification in the context of complex multicellular organisms.

The RLTO model predicts protein overabundance, but is there a clear transcriptional signature? To analyze this question, we define the message threshold $n_{m}\equiv \mu_{m}/o$. (This parameterization is convenient since it is independent of the translation efficiency.) We can then analyze the relation between optimal message number and threshold message number, as shown in Fig. 3A. The model predicts that even for genes that have extremely small threshold message numbers (*e.g.* $n_{m}=10^{-2}$), the optimal message number stays above one message transcribed per cell cycle. Qualitatively, expressing messages below this level is simply too noisy even for proteins needed at the lowest expression levels. (See the blue curve in Fig. 2B corresponding to the protein number distribution of $\mu_{m}=1$.) The model therefore predicts a lower floor on transcription for essential genes of one message per cell cycle.

### A lower threshold is observed for message number.

To identify a putative transcriptional floor, we first analyzed the transcriptome in *Escherichia coli*. We hypothesize that cells must express essential genes above the one-message threshold for robust growth. The distinction between essential and nonessential genes is critical in this context, since nonessential genes can be inducibly expressed. For instance, in *E. coli*, the *lac* operon is repressed in the absence of lactose and therefore need not satisfy the one-message threshold. The transcriptional threshold is only hypothesized to apply to genes whose products are required to maintain cell fitness under the measured conditions.

We generated histograms for *E. coli* growing rapidly on rich media for these two classes of genes. (See Supplementary Material Sec. C.) The message numbers for *nonessential* genes are widely distributed, with a significant fraction of genes falling below the one-message threshold; however, only one *essential* gene is expressed below the one-message threshold (0.3% of essential genes). (See Fig. 3B.) The threshold is not sharp, but rather a smooth depletion relative to a median of 18 messages per cell cycle. This observation is consistent with the predictions of the RLTO model.

To further test this prediction, we then analyzed *E. coli* transcription under slow-growth conditions. Since these cells are less transcriptionally active, we hypothesized that this analysis would constitute a more stringent test of the one-message rule. (See Supplementary Material Sec. C.) To our surprise, although the transcription rate is indeed reduced in slow growth, the essential gene message numbers still satisfy the one-message rule (with a two gene exception, 0.7%), again consistent with the predictions of the RLTO model. (See Fig. 3D.)

Next, we analyzed eukaryotic transcriptomes in *Saccharomyces cerevisiae* (yeast) and *Homo sapiens* (human). (See Supplementary Material Sec. C.) For yeast, there is a well-defined notion of essential genes [19]. As predicted, yeast essential genes obey the one-message threshold (with two exceptions, 0.2%). (See Fig. 3D.) The interpretation is less clear-cut in human cells: An essential gene classification has been generated in the context of proliferation in cell culture [20]. In order to try to capture a generic picture, we average the human transcriptome of cell types. We find that the vast majority of essential genes obey the one-message rule; however, there are significantly more genes that break the rule (81 genes, 8%) than in the other organisms.

### Message number distribution is conserved.

To what extent is this human data consistent with the RLTO model? For human cells, our test of the one-message rule is too simplistic in two respects: (i) We ignore the significant transcriptional differences associated with distinct cell types and (ii) the essential gene classification itself is defined by the ability of mutants or knockdowns to proliferate in cell culture; in marked contrast to the *in vivo* context where cell proliferation is tightly regulated [20]. Due to these subtleties, we decided to take a complementary approach: We considered the distribution of three different transcriptional statistics for each gene: transcription rate, cellular message number, defined as the average number of messages instantaneously, and message number ($\mu_{m}$), defined as the number of messages transcribed in a cell cycle. (See Supplementary Material Sec. C.) The RLTO model predicts a one-message threshold with respect to message number, but not the other two statistics. We therefore predict that the message number distributions in each organism (*E. coli*, yeast, and human) should align for low expression genes with respect to message number, but not for the other two transcriptional statistics. Consistent with the predictions of the RLTO model, there is a striking alignment of message number for essential genes between all three model organisms and growth conditions for message number. (See Fig. 3D.) This alignment is non-trivial: It is not observed with respect to other transcriptional statistics (Fig. 3C and Supplementary Material Fig. 3). The observed alignment is consistent with a conserved transcriptional regulatory program from *E. coli* to human, and we hypothesize that the rationale for this alignment is the abutment of the lowest essential-gene transcription levels in each organism against the one-message threshold.

### Translation efficiency is predicted to increase with transcription.

**FIG. 4.**
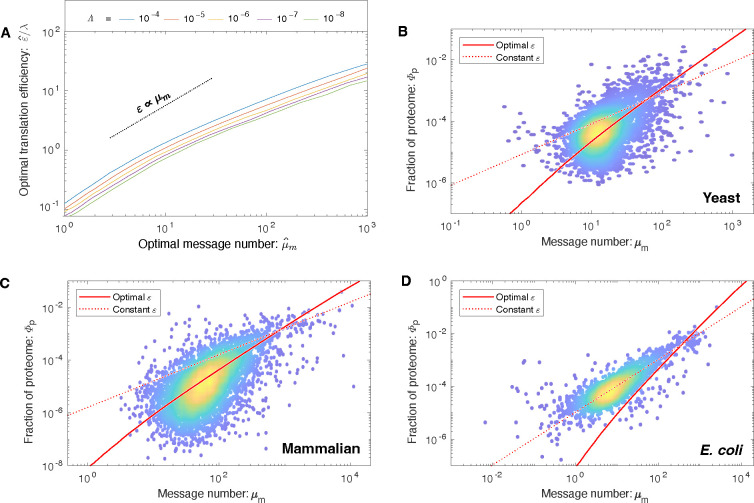
How are transcription and translation balanced? **Panel A: The RLTO model predicts load balancing.** The ratio of the optimal translation efficiency $\left(\overset{ˆ}{\varepsilon }\right)$ to the message cost (λ) is roughly independent of the relative load (Λ). The translation efficiency *ε* is predicted to be roughly proportional message number $\mu_{m}$. **Panel B: RLTO predicts the protein-message-abundance relation in yeast.** The observed proteome fraction is compared to two models: the RLTO optimal model (solid red line) and constant-translation-efficiency model (dotted red line). Both models make parameter-free predictions. The RLTO optimum predicts the global trend. (Data from Ref. [21].) **Panel C: Mammalian proteome fraction.** The RLTO prediction (solid) is superior to the constant-translation-efficiency prediction (dashed). **Panel D: *E. coli* proteome fraction.** In contrast, the constant-translation-efficiency prediction (dashed) is superior to RLTO prediction (solid).

What does the RLTO model predict about how the cell should balance the gene expression process between transcription and translation? Minimizing transcription (at fixed protein abundance) reduces the metabolic load; however, it decreases robustness. Growth rate maximization balances these two costs. Quantitatively, the maximization of the growth rate (Eq. 1) with respect to the translation efficiency can be performed analytically, predicting the optimal translation efficiency, shown in Fig. 4A. We provide an exact expression in the Supplementary Material Sec. A 4; however, an approximate expression for the translation efficiency is more clearly interpretable:

(3) $$\overset{ˆ}{\varepsilon }\approx 0.1\;\lambda {\overset{ˆ}{\mu }}_{m}.$$

The optimal translation efficiency has two important qualitative features for central dogma regulation. The first prediction is that as the message cost (λ) rises, the optimal translation efficiency ($\overset{ˆ}{\varepsilon }$) increases in proportion while the message number decreases. We present evidence for this prediction in the Supplementary Material Sec. A 11.

The second prediction is that the optimal translation efficiency is also approximately proportional to message number $\left(\overset{ˆ}{\varepsilon }\propto \mu_{m}\right)$. Therefore, the RLTO model predicts that low expression levels should be achieved with low levels of transcription and translation, whereas high-expression genes are achieved with high levels of both. We call this relation between optimal transcription and translation the *load balancing principle*. The most direct test of load balancing is measuring the protein-message abundance relation. Due to load balancing, the RLTO model predicts protein number (and proteome fraction) to scale like:

(4) $${\overset{ˆ}{\mu }}_{p}\propto {\overset{ˆ}{\mu }}_{m}^{2},$$

whereas a constant-translation-efficiency model has linear scaling $\left(\mu_{p}\propto \mu_{m}\right)$ Computing proteome fraction, rather than protein number, results in a parameter-free prediction. (See Supplemental Sec. A12.)

### Load balancing is observed in eukaryotic cells.

To test the RLTO predictions, we compare observed proteome measurements in three evolutionarily divergent species, *E. coli* [22], yeast [21] and mammalian cells [23], to two models: the RLTO and the constant-translation-efficiency models. The results of the parameter-free predictions are shown in Fig. 4BCD for each organism. The RLTO model clearly captures the global trend in the proteome-fraction message-number relation in eukaryotic cells and a direct fit to a power law with an unknown exponent is consistent with Eq. 4 (Supplementary Material Sec. D 4 c).

In *E. coli*, the constant-translation-efficiency model better describes the data. Why does this organism appear not to load balance? In the supplementary material, we demonstrate that the observed translation efficiency is consistent with the RLTO model, augmented by a ribosome-per-message limit. Hausser *et al.* have proposed just such a limit, based on the ribosome footprint on mRNA molecules [10]. (See Supplementary Material Sec. A 17.) Although this augmented model is consistent with central dogma regulation in *E. coli*, it is not a complete rationale. This proposed translation-rate limit could be circumvented by increasing the lifetime of *E. coli* messages, which would increase the translation efficiency. A more in-depth analysis specific to *E. coli* is needed to understand why the observed message lifetime is so short.

### RLTO model predicts observed noise in yeast.

Although the protein fraction measurements support the RLTO predictions for the translation efficiency in eukaryotic cells, these measurements do not provide a compelling rationale for why load balancing maximizes the growth rate. To understand its rationale, we explore its implications for noise.

**FIG. 5.**
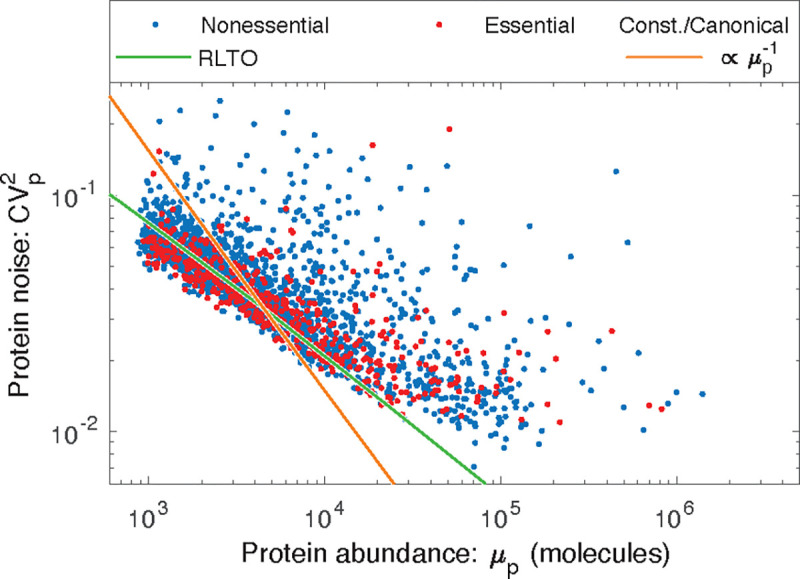
RLTO predicts the magnitude of noise in yeast. The observed gene expression noise is yeast is shown for essential and nonessential genes. Two protein-message abundance models are compared to the data: The RLTO model (green) versus the constant-translation-efficiency (canonical model, orange). The RLTO model predicts both the magnitude of the noise, as well as its scaling with protein abundance. The reduced slope of the RLTO model is the consequence of load balancing, which reduces the noise for the noisiest, low-expression genes. (Data from Ref. [8].)whereas a constant-translation-efficiency model has linear scaling ($\left(\mu_{p}\propto \mu_{m}\right)$. Computing proteome fraction, rather than protein number, results in a parameter-free prediction. (See Supplemental Sec. A 12.)

In a typical biological context, $\mu_{m}\ll \varepsilon$ and as a result, noise production is dominated by the transcription step of the gene expression process [12, 13]. (A table of central dogma parameters for each model organism appears in the Supplementary Material Tab. S2.) Quantitatively, the Paulsson model predicts that the noise should be inversely related to the message number [12, 13]:

(5) $${CV}_{p}^{2}=\frac{ln\;2}{\mu_{m}},$$

however, it is the relation between mean protein abundance $\mu_{p}$ and noise $\left({CV}_{p}^{2}\right)$ which is typically reported [8, 9]. Based on the scaling of the optimal translation efficiency with the message number in eukaryotic cells (Eq. 3), we find the protein number to scale with message number (Eq. 4), which predicts that noise should scale with protein abundance ${CV}_{p}^{2}\propto \mu_{p}^{-1/2}$ in yeast (see Supplementary Material Sec. D 1); however, due to the observed absence of translation-efficiency scaling in bacteria, the noise should scale as ${CV}_{p}^{2}\propto \mu_{p}^{-1}$ in bacteria, as observed [9]. Does the yeast noise show the predicted scaling? The parameter-free RLTO noise prediction closely matches the observed noise in both magnitude and scaling, as shown in Fig. 5.

### Reducing noise is the rationale for load balancing.

This noise analysis also provides a conceptual insight into the rationale for load balancing. The load balanced (RLTO-green) and constant-translation-efficiency (orange) predictions for the noise are shown in Fig. 5. Load balancing results in decreased noise for low-expression, noisy genes over what is achieved with constant translation efficiency. This decreased noise is predicted to increase growth robustness. In principle, the noise could be reduced further by tipping the balance even more towards transcription; however, the RLTO model predicts that this approach is too metabolically costly, and the optimal strategy is that observed for noise scaling in yeast.

## DISCUSSION

### What are the biological implications of noise?

Many important proposals have been made, including bet-hedging strategies, the necessity of feedback in gene regulatory networks, *etc.* [7]. Our model suggests that overcoming cell-to-cell variation may fundamentally reshape the metabolic budget: Typically, proteins constitute 50–60% of the dry mass of the cell [5] and therefore overabundance could increase the overall protein budget by a significant factor. Why does the cell tolerate this significant increase in metabolic load above what would be predicted by a resource allocation analysis (*e.g.* [24])? This program dramatically reduces the consequence of stochastic expression of proteins on the rate of single-cell proliferation.

A second source of stochasticity, environmental fluctuations, has been proposed as a rationale for overabundance [25], especially in the context of metabolic genes [26]. In short, cells express protein to hedge against starvation [25] or changes in the carbon source, *etc.* [26]. How does this hypothesis compare to our growth robustness hypothesis? There are some similarities between these environmental-fluctuation models and the RLTO model: In both models, it is a fluctuations-based mechanism that drives overabundance; however, there are important distinctions between the model predictions. In the environmental fluctuation model, there is a trade-off between log-phase fitness and the rapidity of adaptation [25]; whereas in the RLTO model, overabundance corresponds to the log-phase optimum. Organisms experiencing prolonged periods of balanced growth would therefore be expected to reduce overabundance. Furthermore, the environmental fluctuation model most naturally explains overabundance for proteins related to metabolic processes, whereas the RLTO model predicts overabundance generically, dependent only on message number, which appears to be much more consistent with experiments exploring essential-protein depletion [4].

### Implications for nonessential genes.

In our analysis, we have focused on essential genes in order to motivate the growth-threshold in the RLTO model. To what extent do nonessential genes share the same optimization? In support of the proposal that RLTO optima describe nonessential genes is the success of the model in predicting the translation efficiency for all genes, not just essential genes. (See Fig. 4 and Fig. 5.) Furthermore, the definition of a gene as *essential* depends on context: For instance, in the context of *E. coli* growth on lactose, the gene *lacZ* is essential, although it is nonessential on other carbon sources [27]. Under growth conditions where the *lacZ* gene is essential, we predict that LacZ should be overabundant, consistent with observation [26]. Finally, our modeling suggested that RLTO model phenomenology is the results of asymmetry of the cost of under versus overabundance. For nonessential genes whose activity significantly increases fitness, we still expect fitness asymmetry due to the low relative metabolic cost of increased expression. We therefore expect all gene products, most especially those with low expression, to be overabundant, under conditions where their activity increases fitness.

### Implications of overabundance for inhibitors.

The generic nature of overabundance, especially for low-expression proteins, has important potential implications for the targeting of these proteins with small-molecule inhibitors (*e.g.* drugs). For the highest expression proteins, like the constituents of the ribosome, relatively small decreases in the active fraction (*e.g.* a three-fold reduction) are expected to lead to growth arrest [3]. This may help explain why inhibitors targeting translation make such effective antimicrobial drugs. However, we predict that the lowest expression proteins require a much higher fraction of the protein to be inactivated, with the lowest-expression proteins expected to need more than a 100-fold depletion. This predicted robustness makes these proteins much less attractive drug targets [28].

### The principles that govern central dogma regulation.

**FIG. 6.**
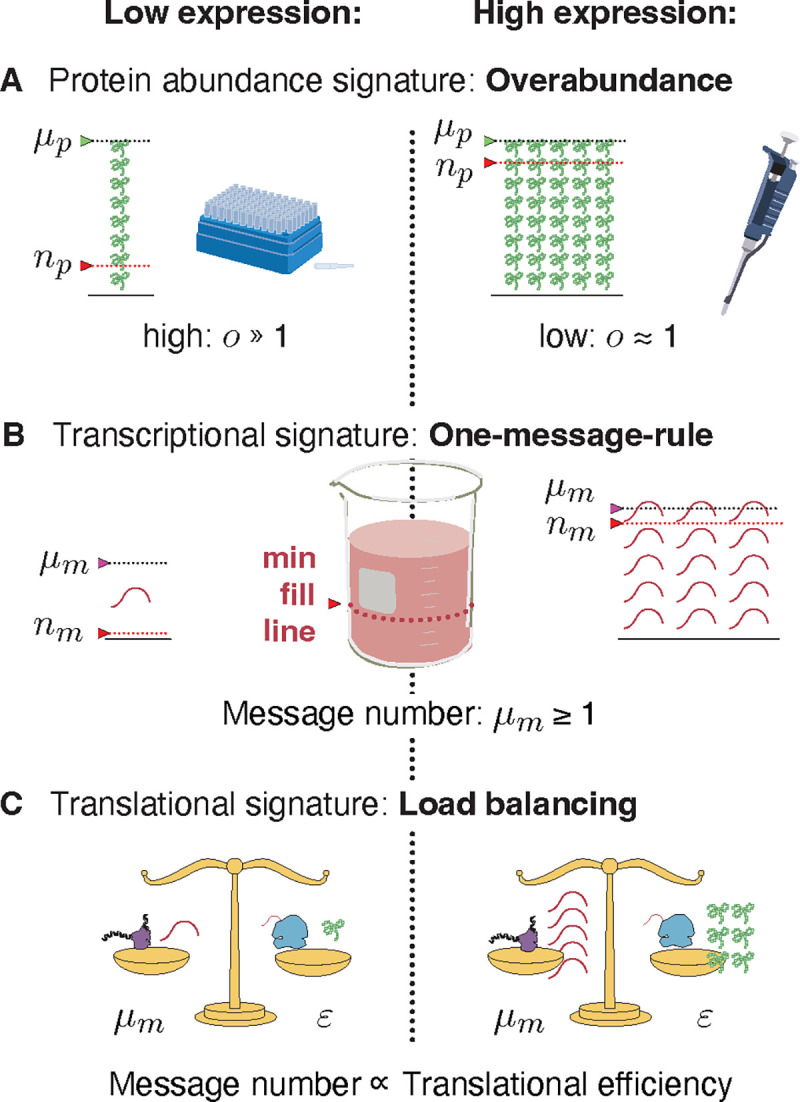
Central dogma regulatory principles. **Panel A: Overabundance.** Low-expression essential genes are expressed with high overabundance; whereas, high-expression essential genes are expressed with low overabundance. Lab supply analogy: Low-cost items that are used stochastically (*e.g.* pipette tips) are purchased in great excess, while the higher cost items that are less stochastic (*e.g.* pipette) are purchased as needed. **Panel B: One-message rule.** Robust expression of essential genes requires them to be transcribed above a threshold of one message per cell cycle. **Panel C: Load balancing.** In eukaryotic cells, optimal fitness is achieved by balancing transcription and translation: The optimal message number is proportional to the optimal translation efficiency. High (low) expression levels are achieved by high (low) levels of transcription followed by high (low) levels of translation per message.

We propose that robustness to noise fundamentally shapes the central dogma regulatory program for all genes and predicts a number of key regulatory principles. (See Fig. 6.) For high-expression genes, load balancing implies that gene expression consists of both high-amplification translation and transcription. The resulting expression level has low overabundance relative to the threshold required for function. In contrast, for essential low-expression genes, a three-fold strategy is implemented: (i) overabundance raises the mean protein levels far above the threshold required for function, (ii) load balancing, and (iii) the one-message rule ensure that message number is sufficiently large to lower the noise of inherently-noisy, low-expression genes. We anticipate that these regulatory principles, in particular protein overabundance, will have important implications, not only for our understanding of central dogma regulation specifically, but for understanding the rationale for protein expression level and function in many biological processes.

## MATERIALS AND METHODS

### RLTO model.

The effect of stochastic cell arrest can be implemented analytically as follows: The probability of growth is the probability that all essential proteins are above threshold, $P_{+}$. The population growth rate k is [29]:

(6) $$\frac{k}{k_{0}}=1+\frac{1}{ln\;2}\ln P_{+},$$

for a population of cells subject to stochastic arrest with probability $1-P_{+}$ per cell cycle where $k_{0}$ is the growth rate of the non-arrested cells. For each gene i, the Paulsson model predicts the protein number CDF in terms of message number $\mu_{m}$ and translation efficiency ε [12]. Assuming the below-threshold probability is small, the probability that the cell is below threshold for gene i is:

(7) $$\ln P_{+,i}=-\gamma (\frac{\mu_{m}}{\ln2},\frac{n_{p}}{\varepsilon \ln2}),$$

where γ is the regularized incomplete gamma function and the CDF of the gamma distribution. (See Supplementary Material Sec. A 2.)

While protein underabundance slows cell growth by the arrest of essential processes, protein overabundance slows growth by increasing the metabolic load. To implement the metabolic-load contribution to cell fitness, we use a minimal model that realizes the metabolic cost of both transcription and translation that is analogous to those previously used in the context of resource allocation (*e.g.* [14]). The metabolic load of transcription and translation of gene i is:

(8) $$\frac{k}{k_{0}}=1-\frac{\lambda +\varepsilon }{N_{0}}\mu_{m},$$

where $k_{0}$ is the growth rate in the absence of the metabolic load of gene $i,\;N_{0}$ is the total cellular metabolic load, and λ is the metabolic message cost. (See the Supplementary Material Sec. A 2 for a detailed development of the model.) The λ-term represents the metabolic cost of transcription and the ε-term represents the metabolic cost of translation of gene i. We define the relative load as $\Lambda \equiv \lambda /N_{0}$ as the ratio of the metabolic load of a single message to the total metabolic cost of the cell. In *E. coli*, we estimate that Λ is roughly 10^−5^ and it is smaller still for eukaryotic cells.

### Data analysis.

We provide a detailed description of the data analysis for the one message rule, loading balancing, and the noise analysis in the Supplementary Material.

## Supplementary Material

Supplement 1

## Data Availability

All data needed to evaluate the conclusions in the paper are present in the paper and/or the Supplementary Materials.
