# Supplementary material for: Noise robustness and metabolic load determine the principles of central dogma regulation
Source: ArXiv. 2024 Jan 8:arXiv:2310.13803v2. Preprint. [Version 2] (PMC10802679)
Supplement: Supplementary file 1 [file NIHPP2310.13803V2-supplement-1.pdf]

Supplementary Material: Noise robustness and metabolic load determine the principles of central dogma regulation

Teresa W. Lo,[1,*] Han Kyou James Choi,[1,*] Dean Huang,[1] and Paul A. Wiggins[1,2,3,†]

[1]*Department of Physics, University of Washington, Seattle, Washington 98195, USA*
[2]*Department of Bioengineering, University of Washington, Seattle, Washington 98195, USA*
[3]*Department of Microbiology, University of Washington, Seattle, Washington 98195, USA*

arXiv:2310.13803v2 [q-bio.MN] 8 Jan 2024

CONTENTS

A. Detailed development of the RLTO model 2
1. Methods: The derivation of the RLTO growth rate 2
a. Metabolic load 2
b. Growth rate with stochastic arrest 3
c. RLTO growth rate 3
d. When the ln approximation is avoided... 4
e. Single-gene equation 4
f. Summary of RLTO parameter values for figures 4
2. Discussion: The fitness landscape of a trade-off. 5
3. Methods: Central dogma optimization 5
a. Optimization of transcription and translation (eukaryotes) 5
b. Optimization of message number only 7
c. Inclusion of the noise floor 7
4. Discussion: Understanding the rationale for overabundance 8
5. Discussion: RLTO predicts larger overabundance in bacteria. 9
6. Discussion: RLTO predicts proteins are buffered to depletion. 9
7. Results: Detailed development of load balancing 9
a. Prediction of the optimal load ratio. 9
b. Measurements of the load ratio 9
8. Results: Translation efficiency is predicted to increase with transcription. 9
9. Results: RLTO predicts that message number responds to message cost 10
10. Results: RLTO predicts the yeast global regulatory response 10
11. Methods: Prediction of the proteome fraction 10
a. Parameter-free prediction of proteome fraction. 10
b. RLTO: proteome fraction 10
c. Constant-translation-efficiency model: proteome fraction 10
d. Sources of experimental data for proteome fraction analysis 11
12. Results: Load balancing is observed in eukaryotic cells 11
13. Discussion: Relation between load balancing and previous results 11
14. Methods: Analysis of translational limits & gene-specific load analysis 13
15. Results: Increased protein-specific cost reduces the optimal translation efficiency. 13
16. Discussion: Translation limits in *E. coli* 14
17. Methods: Estimate of the message cost and metabolic load 14
a. Detailed protocol 15
b. Estimate the message cost and metabolic load in yeast 15
c. Estimate the message cost and metabolic load in human cells 15
d. Estimate of the modified relative load in bacterial cells 15

B. Model robustness & exploring alternatives to RLTO 15
1. Methods: Defining alternative models 15

---

[*] These two authors contributed equally

[†] pwiggins@uw.edu

a. Model 1: Arrest (RLTO) model 16
b. Model 2: Slow-Growth model
c. Model 3: Symmetric model 16
2. Results: Overabundance is a robust prediction 16

C. Quantitation of central dogma parameters for one-message-rule 16
1. Methods: Selection of central dogma parameter estimates 16
a. *E. coli* data 17
b. Yeast data 17
c. Human data 18
2. Methods: Quantitative estimates of central dogma parameters 18
a. Estimating the *cellular message number*: $\mu_{m/c}$ 18
b. Estimating the *transcription rate*: β_m 18
c. Estimating the *message number*: μ_m 18
3. Results: Histograms of central dogma transcriptional statistics 18
4. Discussion: *E. coli* essential genes below the one-message-rule threshold 18

D. Analysis of gene-expression noise 18
1. Methods: Detailed description of the noise model 19
a. Telegraph model: Stochastic kinetic model for central dogma. 19
b. Statistical model for protein abundance. 20
c. Gillespie simulation of the telegraph model 21
d. Gamma function and distribution conventions 21
2. Results: RLTO model predicts non-canonical noise scaling 21
3. Methods: Analysis of gene expression noise 22
a. Hypothesis test I 22
b. Hypothesis test II 22
c. Maximum likelihood estimates of the parameters 23
d. Details: Statistical details MLE estimate of the variance 23
4. Results: Non-canonical noise scaling is observed in yeast. 23
5. Methods: Parameter-free prediction of noise from protein-message relation. 23
a. Estimating *protein number* (μ_p) for the noise analysis 23
b. Empirical models for yeast gene expression 24
c. Empirical model for protein number 24
d. Empirical model for message number 24
e. Empirical model for translation efficiency 24
f. Empirical model for the coefficient of variation 24
6. Results: Parameter-free prediction of noise-abundance in yeast 25
7. Discussion: Implications of noise 25

References 26

Appendix A: Detailed development of the RLTO model

In this section, we provide a detailed development of the RLTO model. First, we provide a derivation of the growth rate as a function of the model parameters (Sec. A 1) as well as other methods (Secs. A 3, A 11, A 14, and A 17). For each of the results discussed in the main paper, we provide more detailed analyses, which include both supplemental results (Secs. A 7, A 8, A 10, A 12, and A 15) that support the story described in the main paper, as well as supplemental discussions (Secs. A 2, A 4, A 5, A 6, A 13, and A 16).

1. Methods: The derivation of the RLTO growth rate

a. Metabolic load

In the absence of cell cycle arrest, we will assume that the cell cycle duration τ is proportional to the overall metabolic load of the messages, proteins, and other cellular components [1]. Focusing on a particular gene:

$$\frac{\tau}{\delta\tau} = N_0 = N_0' + \lambda\mu_m + \mu_p, \qquad \text{(A1)}$$

where $\delta\tau$ is a constant with units of time, N_0 is the total metabolic load (in protein equivalents), N_0' is the total metabolic load of everything but gene i (in units of protein equivalents), and λ is the message cost, the total

metabolic load associated with an mRNA molecule relative to a single protein molecule of the gene product. We will assume the parameters N_0, $\delta\tau$ and λ are global (*i.e.* gene independent), while the message number μ_m and translation efficiency ε, and therefore the protein number:

$$\mu_p = \varepsilon \mu_m, \tag{A2}$$

are gene specific. The total metabolic load in protein equivalents is:

$$N_0 \equiv L_0 + \sum_i \{(\lambda + \varepsilon)\mu_m\}_i, \tag{A3}$$

where L_0 is the load of non-protein and message cellular contributions and the sum runs over all genes. The metabolic load of transcription for gene i is $\lambda\mu_{m,i}$ and for translation, $\mu_{p,i} = \varepsilon_i \mu_{m,i}$.

Although the global parameters N_0, $\delta\tau$ and λ provide an intuitive representation of the model, the relative growth rate depends on fewer parameters. Let k and k_0 be the growth rates in the presence and absence of the metabolic load of gene i. The relative growth rate is:

$$\tfrac{k_0}{k} = 1 + (\Lambda + E)\mu_m, \tag{A4}$$

where we have introduced two new reduced parameters: the relative load, defined as $\Lambda \equiv \lambda/N_0$, represents the ratio of the metabolic load of a single message to the total load and the relative translation efficiency, defined $E \equiv \varepsilon/N_0$, which is the ratio of the number of proteins translated per message to the total metabolic load N_0. (Note that due to the high multiplicity $N_0 \gg (\lambda+\varepsilon)\mu_m$, we can ignore the distinction between N_0 and N_0' in the demoninator.) If we neglect the difference between the total metabolic load and the number of proteins, the proteome fraction for gene i is:

$$\Phi_p = E\mu_m. \tag{A5}$$

Both reduced parameters, Λ and E are extremely small. In *E. coli*, we estimate that both Λ and E are roughly 10^{-5} and they are smaller still for eukaryotic cells. (See Sec. A 17.)

b. Growth rate with stochastic arrest

For completeness, we provide a derivation of the growth rate with stochastic cell-cycle arrest that we have previously described [2]. Starting from the exponential mean expression for the population growth rate [2]:

$$k = \tfrac{\ln 2}{\overline{T}}, \tag{A6}$$

where k is the population growth rate and

$$\overline{T} \equiv -\frac{1}{k} \ln \mathbb{E}_T \exp(-kT), \tag{A7}$$

is the exponential mean where T is the stochastic cell cycle duration [2, 3].

Let P_+ be the probability of growth. When the cells are growing, the cell cycle duration is given by the metabolic load cell-cycle duration τ (Eq. A1). Evaluating the expectation gives:

$$\ln 2 = -\ln P_+ + k\tau, \tag{A8}$$

which can be rearranged to give an expression for the population growth rate k:

$$k = \tau^{-1} \ln 2P_+. \tag{A9}$$

As expected, the growth rate goes down as the probability of growth P_+ decreases, stopping completely at $P_+ = \frac{1}{2}$.

c. RLTO growth rate

In the RLTO model, we will assume the probability of growth is the probability that all essential protein numbers are above threshold. We will further assume that each protein number is independent, and therefore:

$$P_+ = \prod_{i\in\mathscr{E}} \Pr\{N_p > n_p\}_i, \tag{A10}$$

where $\mathscr{E}$ is the set of essential genes. Clearly, this assumption of independence fails in the context of polycistronic messages. We will discuss the significance of this feature of bacterial cells elsewhere, but we will ignore it in the current context.

As we will discuss, the probability of arrest of any protein i to be above threshold is extremely small. It is therefore convenient to work in terms of the CDFs which are very close to zero:

$$\ln P_+ = \sum_{i\in\mathscr{E}} \ln\left(1 - \Pr\{N_p < n_p\}_i\right), \tag{A11}$$

$$\approx -\sum_{i\in\mathscr{E}} \Pr\{N_p < n_p\}_i, \tag{A12}$$

$$= -\sum_{i\in\mathscr{E}} \left[\gamma\left(\tfrac{\mu_m}{\ln 2}, \tfrac{n_p}{\varepsilon \ln 2}\right)\right]_i, \tag{A13}$$

where γ is the regularized incomplete gamma function and the CDF of the gamma distribution (see D 1 b and D 1 d). Finally, we will be interested in the quantity:

$$\ln\ln 2P_+ \approx \ln\ln 2 + -\sum_{i\in\mathscr{E}} \tfrac{1}{\ln 2}\left[\gamma\left(\tfrac{\mu_m}{\ln 2}, \tfrac{n_p}{\varepsilon \ln 2}\right)\right]_i, \tag{A14}$$

where we have again expanded the natural logarithm in the limit that the CDF is small.

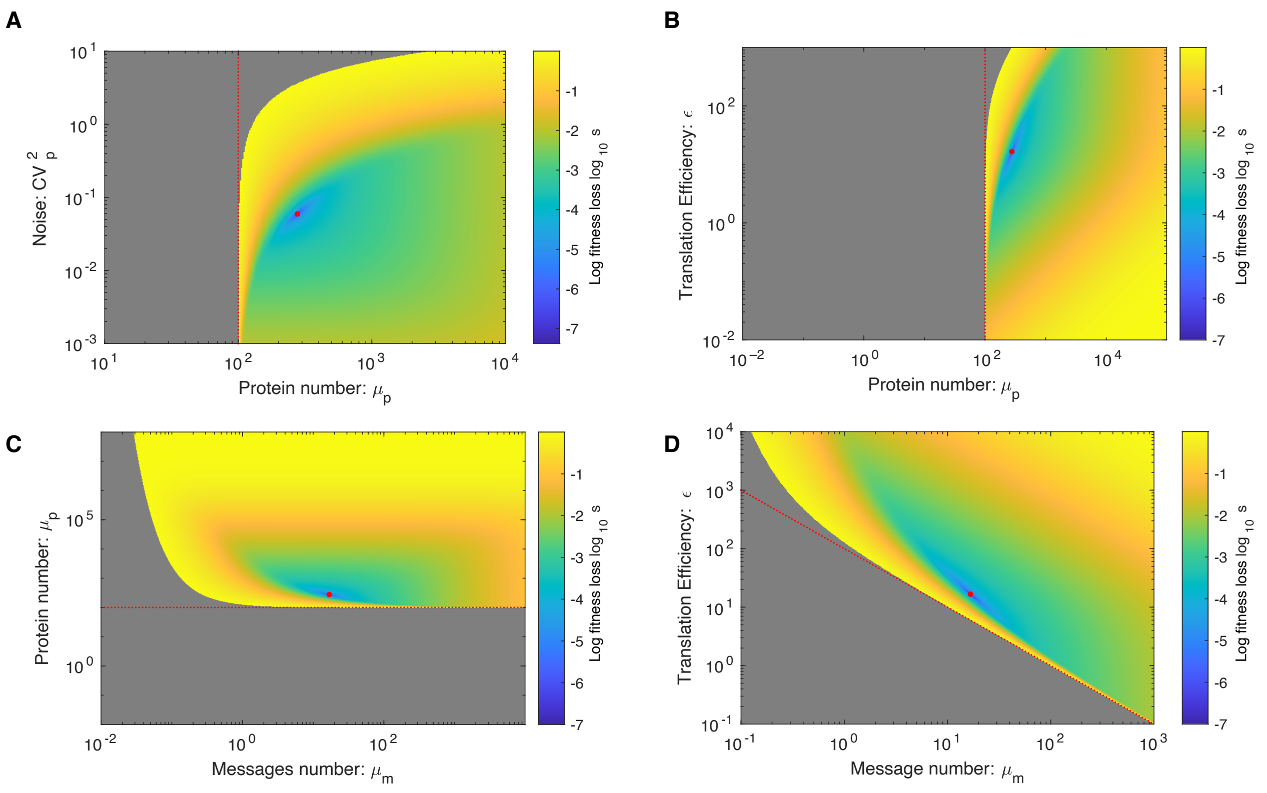

FIG. S1. **Four perspectives on the fitness landscape.** In each landscape, the red circle represents the fitness optimum. The red dotted line represents the mean protein number equal to the protein threshold $n_p = 10^2$. Here fitness is quantified by the log growth rate: $s = \ln k_{\max}/k$. **Panel A: Mean protein number versus noise. Panel B: Mean protein number versus translation efficiency. Panel C: Mean message versus protein numbers. Panel D: Message number versus translation efficiency.**

d. When the ln approximation is avoided...

The approximation discussed in the previous section is extremely well justified at the optimal central dogma parameters; however, there are a set of figures where we cannot use it. In the fitness landscape figures (Fig. S1), we compute the fitness not just at the optimal values but far from them. Here we cannot approximate the natural log and we use the full expression in Eq. A11.

e. Single-gene equation

By combining Eqs. A1, A9, and A14, we can write an expression for the growth rate:

$$\begin{aligned} \ln k &= -\ln\left(L_0 + \sum_i \left[(\lambda+\varepsilon)\mu_m\right]_i\right) + ... \\ &+ \ln\ln 2 - \sum_{i\in\mathscr{E}} \tfrac{1}{\ln 2}\left[\gamma(\tfrac{\mu_m}{\ln 2}, \tfrac{n_p}{\varepsilon \ln 2})\right]_i . \end{aligned} \quad \text{(A15)}$$

We will work in the large multiplicity limit where each of the arrest probabilities is very small. To optimize the expression of gene i we can drop all constant terms and write the simplified expression:

$$\begin{aligned} \ln k &= -\ln\left(N_0' + (\lambda+\varepsilon)\mu_m\right) + ... \\ &+ \ln\ln 2 - \tfrac{1}{\ln 2}\gamma(\tfrac{\mu_m}{\ln 2}, \tfrac{n_p}{\varepsilon \ln 2}) + ..., \end{aligned} \quad \text{(A16)}$$

where we have made the essential gene i subscript implicit and we consider a total load minus gene i:

$$N_0' \equiv L_0 + \sum_{j\neq i} \left[(\lambda+\varepsilon)\mu_m\right]_j , \quad \text{(A17)}$$

interpreted as the metabolic load of all genes but i. However, we will work in the large multiplicity limit where we ignore the distinction between N_0 and N_0'. The fitness landscape for different gene expression parameters is shown from four different perspectives in Fig. S1.

f. Summary of RLTO parameter values for figures

The parameter values for the RLTO model used for each figure in the paper are shown in Tab. S1. All calculations are independent of $\delta\tau$ and L_0.

Figure number and panel:	Relative load: Λ (No units)	Protein cost: λ (molecules^{-1})	Protein threshold: n_p (molecules)	Message number: μ_m (molecules/cell cycle)	Translation efficiency: ε (No units)	Noise floor C_0 (No units)
1F	10^{-5}	10	10^2	Range	Range	0
2A	Range	NA	Range	Local optimum	Local optimum	0
2B	Range	NA	Range	Local optimum	Local optimum	0
3A	Weak dependence (10^{-5})	NA	Range	Local optimum	Local optimum	0
3B	Weak dependence (10^{-5})	NA	Range	Local optimum	Local optimum	0
3C	Weak dependence (10^{-5})	NA	Range	Local optimum	Local optimum	0
S1A	10^{-5}	10	10^2	Range	Range	0
S1B	10^{-5}	10	10^2	Range	Range	0
S1C	10^{-5}	10	10^2	Range	Range	0
S1D	10^{-5}	10	10^2	Range	Range	0
S3A	Range	10^2	Range	Local optimum	Local optimum	0
S3B	Range	10^2	Range	Local optimum	Local optimum	0
S3C	Range	10^2	Range	Local optimum	Local optimum	0
S3D	Range	10^2	Range	Local optimum	Local optimum	0
S4A	Range	10^2	Range	Local optimum	Local optimum	0
S4B	Range	10^2	Range	Local optimum	Local optimum	0
S4C	Range	10^2	Range	Local optimum	Local optimum	0
S4D	Range	10^2	Range	Local optimum	Local optimum	0
S5A	Range	NA	Range	Local optimum	Local optimum	0
S5B	10^{-5}	NA	Range	Local optimum	30	0.1
S6A	Range	NA	Range	Local optimum	Local optimum	0
S7A	Weak dependence (10^{-5})	NA	Range	Local optimum	Local optimum	0
S7B	Weak dependence (10^{-5})	NA	Range	Local optimum	Local optimum	0
S7C	Weak dependence (10^{-5})	NA	Range	Local optimum	Local optimum	0
S7D	Weak dependence (10^{-5})	NA	Range	Local optimum	Local optimum	0
S8	Weak dependence (10^{-5})	NA	Range	Local optimum	Local optimum	0
S10A	Range	NA	Range	Local optimum	Local optimum	0
S14B	Weak dependence (10^{-5})	NA	Range	Local optimum	Local optimum	0

TABLE S1. **RLTO model parameters by figure.** *Range* appears if a range of parameters is used. *NA* appears if the parameter value is irrelevant. *Local optimum* appears if the parameter values in optimized to maximize the growth rate. All calculations are independent of $\delta\tau$ and L_0.

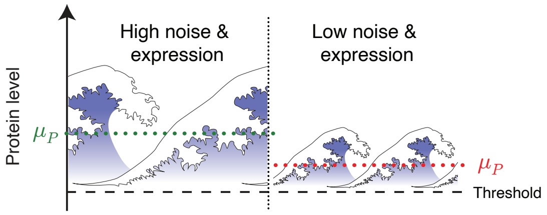

FIG. S2. **Strategies for robustness to noise.** We schematically represent the stochastic protein number as waves. To achieve robust function, protein levels must be above the threshold. Robustness can be achieved by two distinct strategies: (i) increased expression level and (ii) decreased noise.

2. Discussion: The fitness landscape of a trade-off.

The fitness landscape predicted by the RLTO model for representative parameters is shown in Fig. S1. The figure displays a number of important model phenomena: There is no growth for mean protein number μ_p below the threshold number n_p, and for high noise, μ_p must be in significant excess of n_p. Rapid growth can be achieved by the two mechanisms described in Fig. S2: (i) high expression levels (μ_p) are required for high noise amplitude (CV_p^2) or (ii) lower expression levels coupled with lower noise. This trade-off leads to a ridge-like feature of nearly optimal models. The optimal fitness corresponds to a balance between increasing the mean protein number (μ_p) and decreasing noise (CV_p^2). This optimal central dogma program strategy leads to significant overabundance.

3. Methods: Central dogma optimization

a. Optimization of transcription and translation (eukaryotes)

The relative growth rate is:

$$\ln \frac{k}{k_0} = -(\Lambda + E)\mu_m - \frac{1}{\ln 2}\gamma\left(\frac{\mu_m}{\ln 2}, \frac{\phi_p}{E \ln 2}\right), \tag{A18}$$

where γ is the regularized incomplete gamma function, which is the CDF of the gamma distribution and represents the probability of arrest due to gene i. We set the partial derivative of the growth rate with respect to

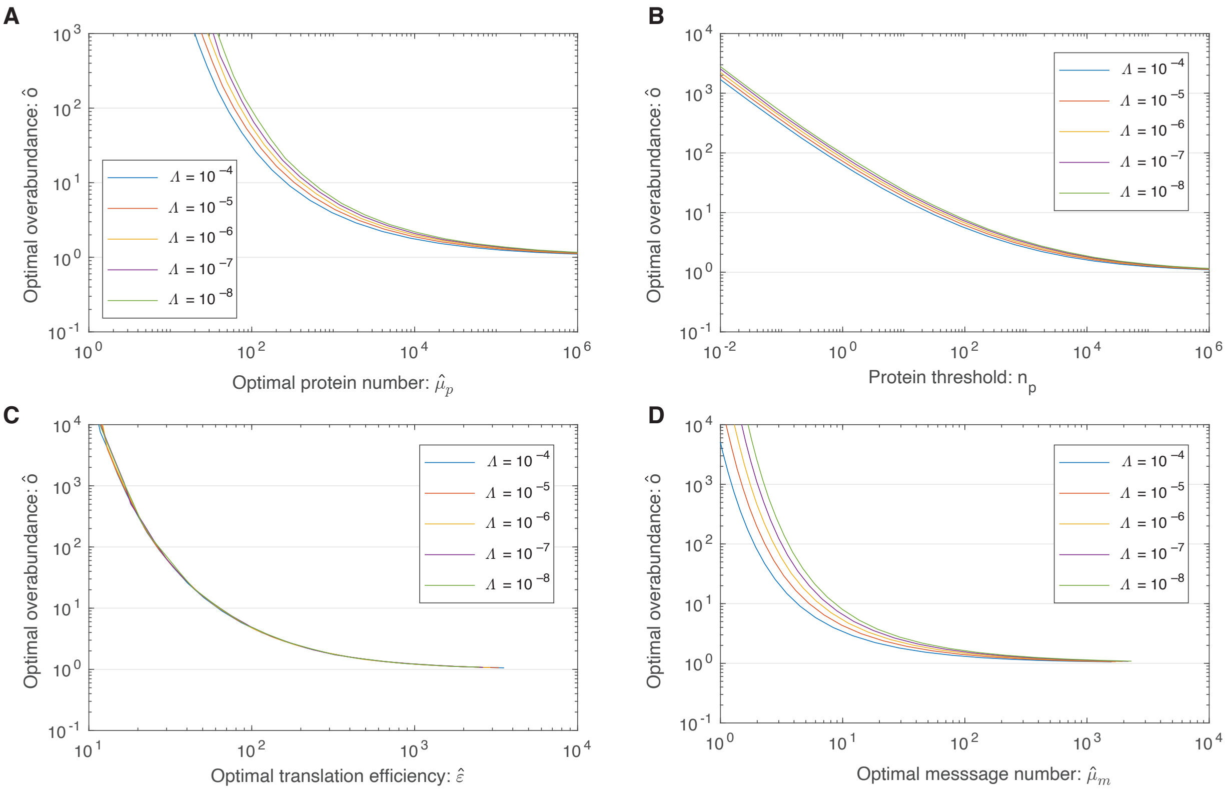

FIG. S3. **Four perspectives on the dependence of optimal overabundance on relative load Λ.** All these calculations are performed for protein cost $\lambda = 100$ in order to give real numbers in molecules per cell. **Panel A: Overabundance as a function of protein number.** Overabundance decreases as protein number increases. These calculations are λ *dependent*. **Panel B: Overabundance as a function of the protein threshold.** Overabundance decreases as protein threshold increases. These calculations are λ *dependent*. **Panel C: Overabundance as a function of translation efficiency.** Overabundance decreases as translation efficiency increases. These calculations are λ *dependent*. **Panel D: Overabundance as a function of message number.** Overabundance decreases as message number increases. These calculations are λ *independent*.

message number equal to zero:

$$0 = -\tfrac{\lambda+\hat{\varepsilon}}{N_0} - \tfrac{1}{(\ln 2)^2}\gamma_{,1}\left(\tfrac{\hat{\mu}_m}{\ln 2}, \tfrac{n_p}{\hat{\varepsilon}\ln 2}\right), \tag{A19}$$

where we use the canonical comma notation to show which argument of γ has been differentiated. Next we differentiate with respect to the translation efficiency to generate a second optimization condition:

$$0 = -\tfrac{\hat{\mu}_m}{N_0} + \tfrac{n_p}{\hat{\varepsilon}^2(\ln 2)^2}\gamma_{,2}\left(\tfrac{\hat{\mu}_m}{\ln 2}, \tfrac{n_p}{\hat{\varepsilon}\ln 2}\right). \tag{A20}$$

We will work in the large multiplicity limit where the overall metabolic load is much smaller than the metabolic load associated with any single gene: $N_0 \gg (\lambda+\hat{\varepsilon})\hat{\mu}_m$. Next, we eliminate the threshold n_p in favor of the optimal overabundance:

$$\hat{o} \equiv \tfrac{\hat{\mu}_p}{n_p} = \tfrac{\hat{\varepsilon}\hat{\mu}_m}{n_p}, \tag{A21}$$

in both Eqs. A19 and A20. Eq. A20 can now be solved for the optimal translation efficiency:

$$\hat{\varepsilon} = \tfrac{N_0}{\hat{o}(\ln 2)^2}\gamma_{,2}\left(\tfrac{\hat{\mu}_m}{\ln 2}, \tfrac{\hat{\mu}_m}{\hat{o}\ln 2}\right). \tag{A22}$$

If we reinterpret γ as the CDF of the gamma distribution, we can rewrite this equation in terms of the gamma distribution PDF:

$$\hat{\varepsilon} = \tfrac{N_0}{\ln 2} p_\Gamma\left(\mu_m \middle| \tfrac{\hat{\mu}_m}{\ln 2}, \hat{o}\ln 2\right), \tag{A23}$$

which will be the optimization equation for the translation efficiency.

To derive the optimization condition for the message number μ_m, we substitute Eq. A22 into Eq. A19:

$$\tfrac{\lambda \ln 2}{N_0} = -\tfrac{1}{\hat{o}\ln 2}\gamma_{,2}\left(\tfrac{\hat{\mu}_m}{\ln 2}, \tfrac{\hat{\mu}_m}{\hat{o}\ln 2}\right) - \tfrac{1}{\ln 2}\gamma_{,1}\left(\tfrac{\hat{\mu}_m}{\ln 2}, \tfrac{\hat{\mu}_m}{\hat{o}\ln 2}\right). \tag{A24}$$

The two terms on the RHS can now be collected as the single partial derivative of message number μ_m:

$$\Lambda \ln 2 = -\partial_{\hat{\mu}_m}\gamma\left(\tfrac{\hat{\mu}_m}{\ln 2}, \tfrac{\hat{\mu}_m}{\hat{o}\ln 2}\right), \tag{A25}$$

where the relative load is $\Lambda \equiv \lambda/N_0$.

The two optimization equations are summarized below:

$$\Lambda \ln 2 = -\partial_{\hat{\mu}_m}\gamma\left(\tfrac{\hat{\mu}_m}{\ln 2}, \tfrac{\hat{\mu}_m}{\hat{o}\ln 2}\right), \tag{A26}$$

$$\tfrac{\hat{E}}{\Lambda} = \tfrac{\hat{\varepsilon}}{\lambda} = \tfrac{1}{\Lambda \ln 2} p_\Gamma\left(\hat{\mu}_m \middle| \tfrac{\hat{\mu}_m}{\ln 2}, \hat{o}\ln 2\right). \tag{A27}$$

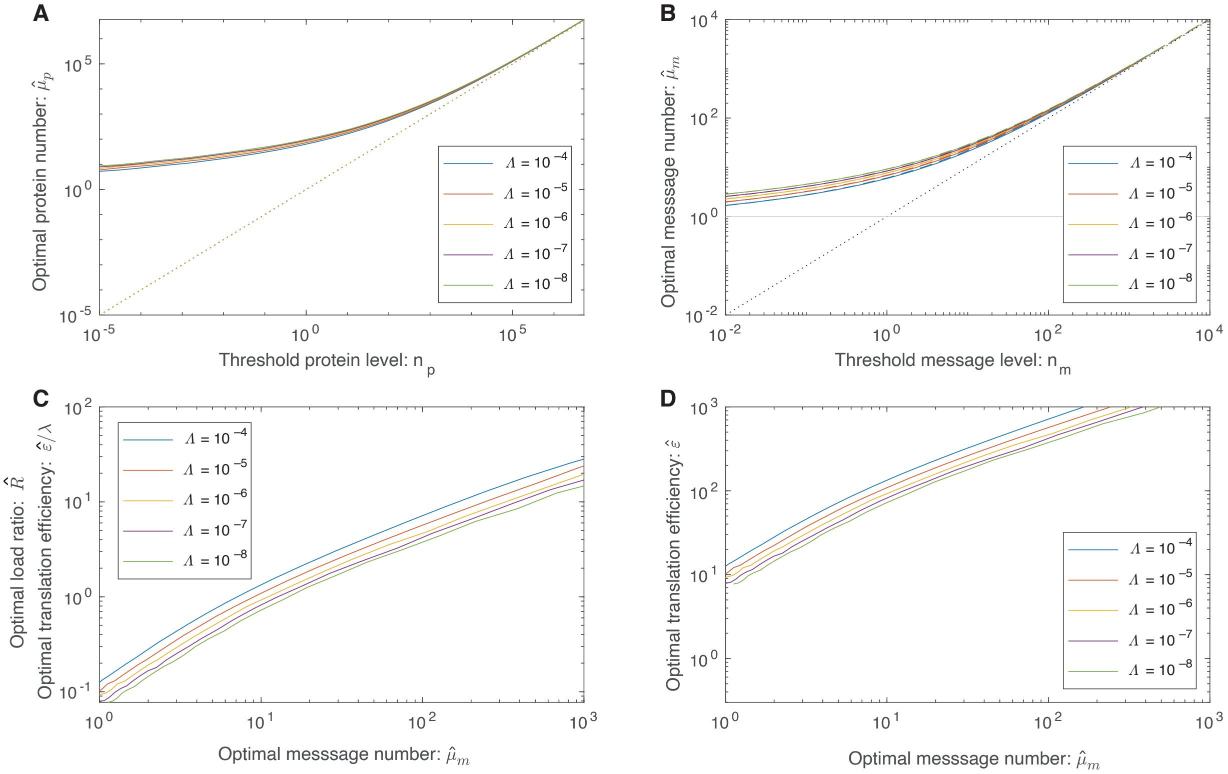

FIG. S4. All these calculations are performed for protein cost $\lambda = 100$ in order to give real numbers in molecules per cell. **Panel A: Protein number versus protein threshold.** At high expression levels, the protein number tracks the protein threshold; however, the one message rule forces the protein number to threshold for low expression levels. These calculations are λ *dependent*. **Panel B: Message number versus message threshold.** At high expression levels, the message number tracks the message threshold; however, the one message rule forces the message number to a threshold close to $\mu_m = 1$ for low expression levels. These calculations are λ *independent*. **Panel C & D: Message number versus translation efficiency.** The optimal translation efficiency grows almost linearly with the optimal message number. The scaled translation efficiency $(\hat{\varepsilon}/\lambda)$ is independent of λ while the translation efficiency $(\hat{\varepsilon})$ is dependent on λ. The ratio ε/λ has a second interpretation: the load ratio R. R is defined as the metabolic cost of translation over transcription of the gene.

The optimal overabundance is shown for a range of relative loads in Fig. S3. The optimal translation efficiency and scaled translation efficiency are shown for a range of relative loads in Fig. S4.

b. Optimization of message number only

Consider the special case of optimizing the message number only at fixed translation efficiency. Eq. A19 is the condition; however, in this case it makes sense to adsorb both the message and protein metabolic load into a single metabolic load. The optimum message number satisfies the equation:

$$\frac{(\lambda+\varepsilon)\ln 2}{N_0} = -[\partial_{\hat{\mu}_m}\gamma(\hat{\mu}_m, \hat{n}_m)]_{\hat{n}_m=\frac{\hat{\mu}_m}{\delta}}. \tag{A28}$$

We define a modified relative load:

$$\Lambda' \equiv \frac{(\lambda+\varepsilon)}{N_0}, \tag{A29}$$

and substitute this into the optimum message number equation:

$$\Lambda' \ln 2 = -[\partial_{\hat{\mu}_m}\gamma(\hat{\mu}_m, \hat{n}_m)]_{\hat{n}_m=\frac{\hat{\mu}_m}{\delta}}. \tag{A30}$$

which is clearly closely related to Eq. A25.

We compare this modified expression to the original for optimum overabundance as a function of message number in Fig. S5A and demonstrate that the two make nearly identical predictions.

c. Inclusion of the noise floor

In bacterial cells, the noise is dominated by the noise floor for high expression genes. Including the noise floor, the coefficient of variation squared is ([4] and Sec. D):

$$\mathrm{CV}_p^2 = \tilde{a}(\mu_m)^{-1} = \frac{\ln 2}{\mu_m} + C_0, \tag{A31}$$

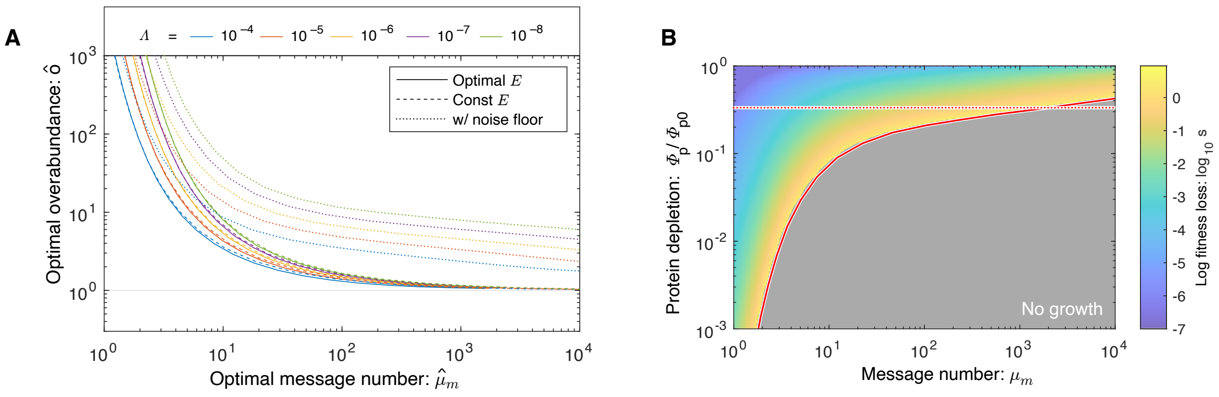

FIG. S5. **Panel A: Overabundance as a function of message number.** In the limit of high message number (μ_m), the noise is small, and the optimal expression level is close to the threshold ϕ_p, leading to minimal overabundance ($o \to 1$); however, as the message number approaches unity, the noise is comparable to the mean, driving vast overabundance ($o \gg 1$). Overabundance increases as the relative metabolic load decreases, and is essentially identical for constant (dashed) versus optimized translation efficiency (solid). To model overabundance in bacterial cells, we included the contribution of the noise floor with fixed translation efficiency (dotted curves), which increases overabundance, especially for high expression genes. **Panel B: Optimal expression levels are buffered.** The predicted fitness loss as a function of protein depletion level and message number for bacterial cells (including the noise floor). Due to the overabundance phenomenon, all proteins are buffered against depletion, but low-expression genes are particularly robust due to higher overabundance. The solid red line represents $1/o$, and predicts the range of depletion values for which cell growth is predicted. The dotted red line represents a three-fold depletion.

where $C_0 = 0.1$ for bacterial cells [4]. In spite of the addition of noise from the noise floor, the observed distribution of protein number is still well described by the gamma distribution [4]; however, we need to modify the statistical parameters to account for the noise floor. (See the definition of the *statistical noise model* in Sec. D 1 b.) The modified gamma parameters are:

$$a = \tilde{a}, \tag{A32}$$
$$\theta = \varepsilon \frac{\mu_m}{\tilde{a}}, \tag{A33}$$

chosen such that the noise is determined by Eq. A31 but the protein number remains:

$$\mu_p = \varepsilon \mu_m, \tag{A34}$$

the product of the message number and translation efficiency.

The qualitative effect of the noise floor is to increase the noise, especially for low-copy messages. Above $\mu_m = 7$ messages, the noise is dominated by the noise floor. Increases in transcription above this point have little effect on reducing the noise. As a consequence, the overabundance stays high, even for high copy messages. We compare this modified expression to the original for optimum overabundance as a function of message number in Fig. S5A and demonstrate that bacterial cells are predicted to have much higher overabundance at high expression levels.

4. Discussion: Understanding the rationale for overabundance

Essential protein overabundance is the signature prediction of the RLTO model. Its mathematical rationale is the highly-asymmetric fitness landscape. To understand why we expect this rationale to be generic, consider the form of the optimization condition for message number:

$$\Lambda \ln 2 = -\frac{\partial}{\partial \hat{\mu}_m} \gamma\left(\tfrac{\hat{\mu}_m}{\ln 2}, \tfrac{\hat{\mu}_m}{\hat{o} \ln 2}\right). \tag{A35}$$

The growth rate is maximized when the probability of slow-growth (*e.g.* arrest) is roughly equal to the relative load of adding one more message. Since the cell makes roughly 10^5 messages per cell cycle, the relative load is extremely small and therefore the probability of slow growth must be as well. Making this probability very small requires vast overabundance for the inherently-noisy, low-expression proteins.

The reason we expect the RLTO model protein abundance predictions to be robust is that we generically expect the fitness cost of overabundance to be small due to the high multiplicity (*i.e.* total number of genes); whereas, the fitness cost of arrest of essential processes is very high.

Should this strategy surprise us? No. We routinely use this strategy in our own lives. For instance, when baking an apple pie, we keep cheap ingredients, needed in small quantities, in vast overabundance (*e.g.* salt); whereas, the more expensive ingredients, needed in higher quantities (*e.g.* apples), are bought only as needed. (See Fig. 4A.)

5. Discussion: RLTO predicts larger overabundance in bacteria.

There are two distinctive features of bacterial cells that could affect the model predictions: (i) the translation efficiency is constant [5] and bacterial gene expression is subject to a large-magnitude noise floor that increases the noise for high-expression genes [4]. The optimization of message number at fixed translation efficiency does result in a slightly modified optimization condition for the message number (Sec. A 3 b); however, the predicted overabundance is only subtly perturbed (Fig. S5A). In contrast, the noise floor increases the predicted overabundance, especially for high-expression proteins. As a result, the RLTO model predicts that the vast majority of bacterial proteins are expressed in significant overabundance. (See Fig. S5A.)

6. Discussion: RLTO predicts proteins are buffered to depletion.

A principle motivation for our analysis is the observation that many protein levels appear to be buffered. To explore the prediction of the RLTO model for protein depletion, we first computed the optimal message numbers and translation efficiencies for a range of protein thresholds. To model the effect of protein depletion, we computed the change in growth rate as function of protein depletion (equivalent to a reduction of the translation efficiency relative to the optimum.) The growth rate is shown in Fig. S5B for the RLTO model with parameters representative of a bacterial cell. (See Sec. A 3 c.)

In general, the RLTO model predicts that protein numbers have very significant robustness (*i.e.* buffering) to protein depletion. This is especially true for low expression proteins that are predicted to have the largest overabundance. For these genes, even a ten-fold depletion leads to very subtle reductions in the growth rate. For a three-fold reduction in the growth rate, only the very highest-expression genes (*e.g.* ribosomal genes) are expected to lead to qualitative phenotypes.

7. Results: Detailed development of load balancing

a. *Prediction of the optimal load ratio.*

The two-stage amplification of the central dogma implies that the expression and noise levels can be controlled independently by the balance of transcription to translation. How does the cell achieve high and low gene expression optimally, and how does this strategy depend on the message cost?

To understand the optimization, we first define the load ratio R for a gene as the metabolic cost of translation relative to transcription:

$$R \equiv \tfrac{\mu_p}{\lambda \mu_m} = \tfrac{\varepsilon}{\lambda}. \tag{A36}$$

In Sec. A 3, we show that the optimal load ratio is:

$$\hat{R} \equiv \tfrac{1}{\Lambda \ln 2}\, p_\Gamma(\hat{\mu}_m | \tfrac{\hat{\mu}_m}{\ln 2}, \tfrac{1}{\delta \ln 2}), \tag{A37}$$

where p_Γ is the PDF of the gamma distribution. The optimal load ratio is shown in Fig. S4C.

The dependence of the optimal load ratio $\hat{R}$ on Λ is extremely weak, but it is strongly dependent on message number. As a result, for low transcription genes ($\mu_m \ll 10$), the metabolic load is predicted to be dominated by transcription; whereas, for highly transcribed genes ($\mu_m \gg 10$), the metabolic load is dominated by translation. These predictions are robust since they are independent of the relative load Λ.

b. *Measurements of the load ratio*

Unfortunately, there is somewhat limited data to which to compare the model. The best source we found was Kafri *et al.* [6] who analyzed the differences in fitness between transcription and transcriptional-and-translation of a fluorescent protein driven by the pTDH3 promoter in yeast. This promoter is one of the strongest in yeast. Based on the RLTO model, we would predict this promoter to have a very high translation efficiency and therefore a large load ratio; however, the translation efficiency is much lower than one would predict based on a global analysis and likewise its load ratio is roughly unity, which based on the smaller than expected translation efficiency is broadly consistent with our expectations. A satisfactory test of this prediction will require larger-scale measurements that probe more representative genes.

8. Results: Translation efficiency is predicted to increase with transcription.

Now that we have defined the optimal load ratio (Eq. A37), the equation for optimal translation efficiency can be written concisely:

$$\hat{\varepsilon} = \lambda \hat{R} \quad \text{or} \quad \hat{E} = \Lambda \hat{R}, \tag{A38}$$

where $\hat{R}$ depends weakly on the relative load Λ. The RLTO model predicts that optimal partitioning of amplification between transcription (gain μ_m) and translation (gain ε) has two important qualitative features: (i) As the message cost (λ) rises, the optimal translation efficiency increases in proportion. (ii) The optimal translation efficiency is also approximately proportional to message number ($\hat{\varepsilon} \propto \mu_m$). (See Fig. S7A.) Therefore, the RLTO model predicts that low expression levels should

be achieved with low levels of transcription and translation, whereas high expression genes are achieved with high levels of both. We call this relation between optimal transcription and translation *load balancing*.

9. Results: RLTO predicts that message number responds to message cost

We will first focus on analyzing the implications of the message cost dependence in Eq. A38. At a fixed load ratio, Eq. A38 clearly implies that the translation efficiency increases as the message cost λ increases; however, the message number (and load ratio) also respond to compensate to changes in λ. To probe the dependence on message cost in an experimentally relevant context, consider optimal message numbers in a reference condition (relative load Λ_0) relative to a second perturbed condition (relative load Λ). The predicted relation between the optimal messages numbers is shown in Fig. S6A. The resulting relation between the optimal message numbers is roughly linear on a log-log plot, predicting the approximate power-law relation:

$$\hat{\mu}_m(\Lambda) \propto \hat{\mu}_m(\Lambda_0)^\alpha, \tag{A39}$$

describing a non-trivial global change in the regulatory program.

10. Results: RLTO predicts the yeast global regulatory response

To test the RLTO predictions, we compared the relative message numbers for yeast growing under phosphate depletion, which increases the message cost [6], to a reference condition [7]. As predicted, the relative transcriptome data was well described by a power law (Eq. A39) and the observed slope was smaller than one: $\hat{\alpha} = 0.837 \pm 0.001$, as predicted by the increased message cost. See Fig. S6B.

The observation of this large-scale regulatory change has an important implication: This response supports a nontrivial hypothesis that the RLTO model not only can predict how the cell is optimized in an evolutionary sense, but can predict global regulatory responses as well.

Note that Metzl-Raz *et al.* [7] also explored conditions that increased the cost of protein; however, here the predictions of the model are ambiguous. The complication arises due to the observed decrease in size of the cells in the experimental condition, which decreases the total metabolic load N_0. As discussed above, the relative load $\Lambda = \lambda/N_0$ is the key determinant in Eq. A39; however, even as the relative cost of transcription λ decreases in the experimental condition, the total metabolic load N_0 also decreases, making no clear prediction about how the relative load Λ changes.

11. Methods: Prediction of the proteome fraction

a. *Parameter-free prediction of proteome fraction.*

We now turn our focus to an analysis of the implications of *load balancing*: the message number dependence of the optimal translation efficiency (Eq. A38). The most direct test of this prediction is measuring the relation between proteome fraction and message number. The RLTO model predicts proteome fraction (Eq. A5):

$$\hat{\Phi}_p = \hat{E}\hat{\mu}_m \propto \hat{\mu}_m^2, \tag{A40}$$

where μ_m is the observed message number and the optimal relative translation efficiency is predicted by Eq. A38. The proportionality is only approximate but gives important intuition for how protein number depends on message number in the RLTO model, in contrast to a constant-translation-efficiency model: $\Phi_p \propto \mu_m$. To compare these predictions to protein abundance measurements, we will renormalize the protein fraction to be defined relative to total protein number rather than N_0. This renormalization eliminates the Λ dependence to result in a parameter-free prediction of the proteome fraction.

b. *RLTO: proteome fraction*

Starting from Eq. A68, clearly:

$$\hat{\mu}_p \propto \hat{\mu}_m \, p_\Gamma(\hat{\mu}_m | \tfrac{\mu_m}{\ln 2}, \hat{o}\ln 2), \tag{A41}$$

which can be used to predict the proteome fraction:

$$\hat{\Phi}_{pi} \equiv \tfrac{\hat{\mu}_{pi}}{\sum_j \hat{\mu}_{pj}}, \tag{A42}$$

where the second subscript is the gene index. To predict the proteome fraction, we computed the proportionality constant C:

$$C \equiv \textstyle\sum_i \left[\mu_m p_\Gamma(\mu_m | \tfrac{\mu_m}{\ln 2}, o\ln 2)|_{o=\hat{o}(\mu_m)}\right]_i, \tag{A43}$$

where the message numbers μ_{mi} for gene i are the experimentally observed message numbers, the implicit o_i values are predicted by the RLTO model (Eq. A25) for message number μ_{mi} and the sum index i runs over all genes. The predicted optimal proteome fraction is:

$$\hat{\Phi}_{pi} = C^{-1}\left[\mu_m p_\Gamma(\mu_m | \frac{\mu_m}{\ln 2}, o\ln 2)|_{o=\hat{o}(\mu_m)}\right]_i, \tag{A44}$$

which generates the predicted solid curves shown in Fig. S7BCD.

c. *Constant-translation-efficiency model: proteome fraction*

For the constant translation efficiency model, we define the normalization:

$$C' \equiv \sum_i \mu_{mi}, \tag{A45}$$

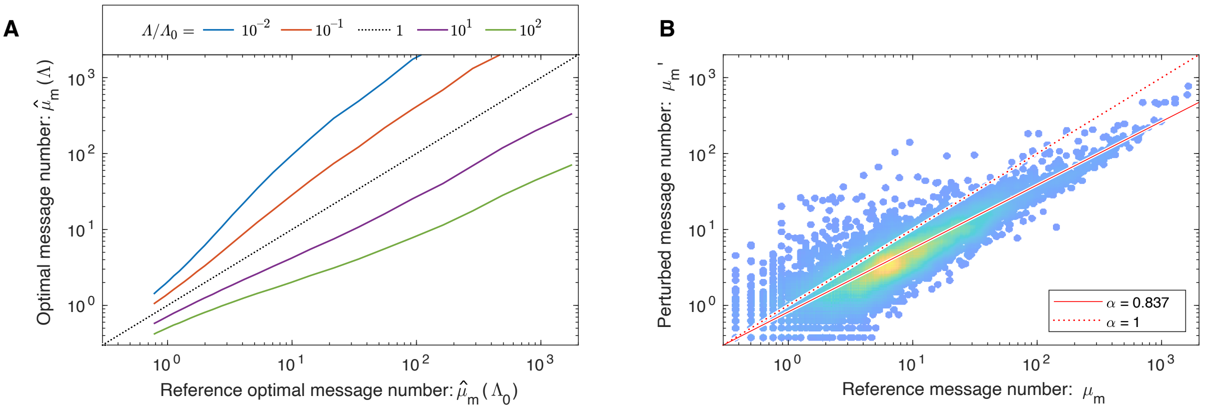

FIG. S6. **Panel A: Message number decreases with increased relative load** Λ**.** The optimal message number responds to changes in the message cost. The RLTO model predicts an approximate power-law relation (linear on a log-log plot) between message numbers. **Panel B: A power-law relation is observed.** To test whether central dogma regulation would adapt dynamically as predicted, we analyzed the relation between the yeast transcriptome under reference conditions and phosphate depletion (perturbed), which increases the message cost [6]. (Data from Ref. [7].) As predicted by the RLTO model, a global change in regulation is observed, which generates a power-law relation with scaling exponent $\alpha = 0.837 \pm 0.01$. The observed exponent is smaller than one, as predicted by an increased relative load Λ.

and the predicted proteome fraction is:

$$\Phi'_{pi} = C'^{-1}\mu_{mi}, \quad \text{(A46)}$$

which generates the predicted dotted curves shown in Fig. S7BCD.

d. Sources of experimental data for proteome fraction analysis

For *E. coli* data, the protein abundance data was generated by mass spec measurements and the message abundance data was from [5]. For the yeast data, the protein abundance data is measured by mass spec and message abundances are determined by [8]. For the mammalian data, we used mouse data. The protein abundance data is measured by mass-spec and message abundances are determined by [9].

We estimated the message number μ_m as described in Sec. C 2 c. For the mouse data, the study provided message lifetimes, the cell cycle duration and abundances in molecules per cell [9]. For the *E. coli* and yeast, the total number of proteins, messages *etc*, cell cycle duration and message lifetimes for each organism and their sources are described in Tab. S2.

12. Results: Load balancing is observed in eukaryotic cells

A non-trivial prediction of the RLTO Model is that translation efficiency and message number should be roughly proportional. Qualitatively, this strategy allows expression levels to be increased while distributing the added metabolic load between transcription, which reduces noise, and translation, which does not affect the noise. We predict the optimal translation efficiency versus message number which matches the observations in eukaryotic cells (Fig. S7BC). However, in *E. coli*, the translation efficiency and message number are *not* strongly correlated (Fig. S7D). Why does this organism appear not to load balance? We demonstrate that the observed translation efficiency is consistent with the RLTO model, augmented by a ribosome-per-message limit. (See Sec. A 14.) Hausser *et al.* have proposed just such a limit, based on the ribosome footprint of mRNA molecules [10]. (See Sec. A 16.) Although this augmented model is consistent with central dogma regulation in *E. coli*, it is not a complete rationale. This proposed translation-rate limit could be circumvented by increasing the lifetime of *E. coli* messages which would increase the translation efficiency. Why the message lifetime is as short as observed will require a more detailed *E. coli*-specific analysis.

13. Discussion: Relation between load balancing and previous results

Hausser *et al.* have previously performed a more limited analysis of the trade-off between metabolic load and gene-expression noise [10]. In this section, we will provide some more context into the differences between the two approaches.

Hausser *et al.* assume a symmetric (not an asymmetric) fitness landscape and consider only the metabolic cost of transcription (but not translation). Their model depends on two (not one) gene-specific parameters: an optimal protein number and a sensitivity, which defines the curvature of the fitness [10]. The authors maximize fitness with respect to the transcription rate (but not

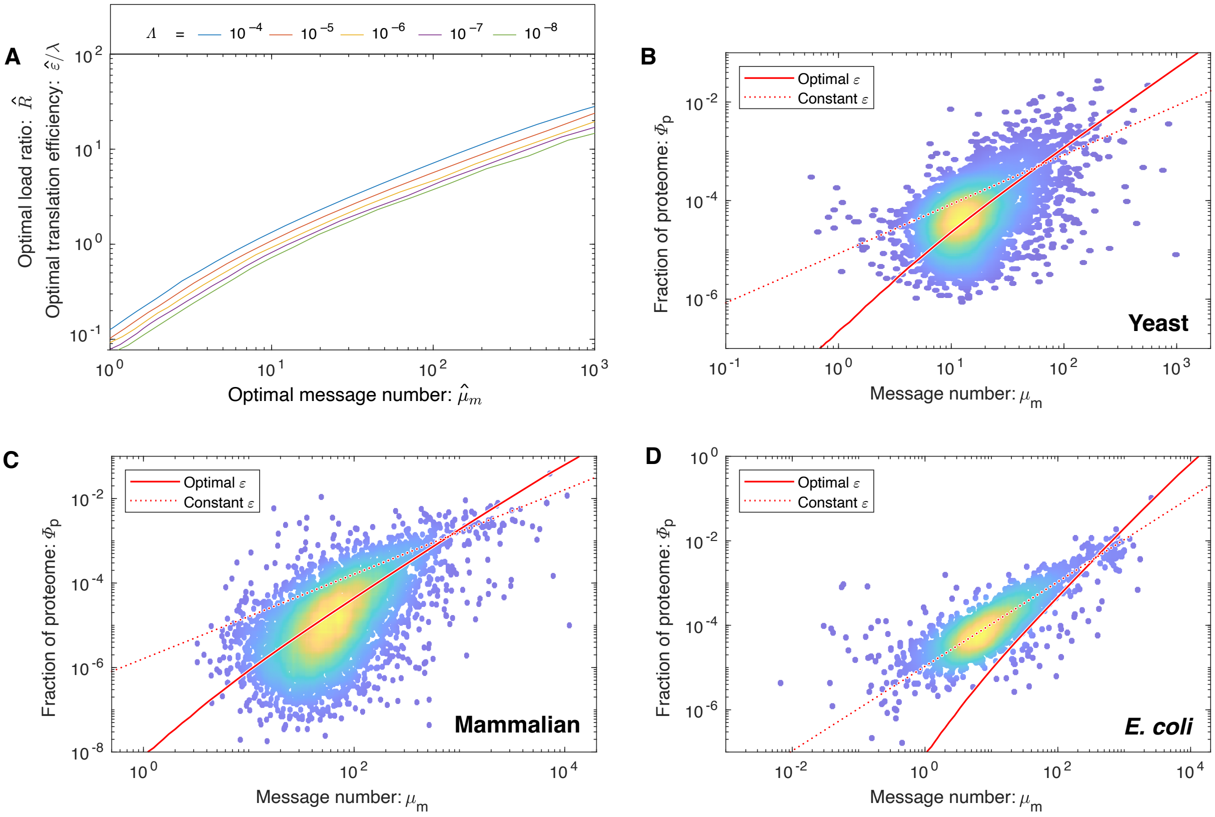

FIG. S7. **Panel A: Load balancing: the optimal translation efficiency increases with message number and cost.** The ratio of the optimal translation efficiency ($\hat{\varepsilon}$) to the message cost (λ) is roughly independent of the relative load (Λ); therefore, the optimal translation efficiency is expected to be proportional to the message cost. The translation efficiency is also roughly proportional to the optimal message number $\hat{\mu}_m$. The ratio ε/λ has a second interpretation: the load ratio R. R is defined as the metabolic cost of translation over transcription of the gene. **Gene proteome fraction versus message number for three model systems.** Two models for relation between gene proteome fraction and message number are compared with observations: The RLTO model makes a parameter-free prediction of the optimal relation (*optimal*, solid red line). A second competing model is constant translation efficiency (*constant*, dotted red line). **Panel B: Yeast proteome fraction.** The RLTO prediction (solid) is superior to the constant-translation-efficiency prediction (dashed). **Panel C: Mammalian proteome fraction.** The RLTO prediction (solid) is superior to the constant-translation-efficiency prediction (dashed). **Panel D: *E. coli* proteome fraction.** In contrast, the constant-translation-efficiency prediction (dashed) is superior to RLTO prediction (solid).

the translation rate) and the condition they derive depends on the two (not one) unknown, gene-specific parameters. As a result, this condition is not predictive of global regulatory trends without non-trivial, gene-specific measurements or assumptions about the unknown sensitivity.

The Hausser model assumes that the growth rate has the form:

$$k = k_0 - \tfrac{1}{2}|k''|(N_p - \mu_p)^2 - k_0\Lambda\mu_m, \tag{A47}$$

where k is the growth rate, and we have rewritten the form of the fitness to better match our own definitions. Here k'' is the second derivative of the growth rate at the optimal protein number μ_p and N_p is the stochastic protein number. If we take the expectation with respect to the protein number, we get:

$$k = k_0 - \tfrac{1}{2}|k''|\sigma_p^2 - k_0\Lambda\mu_m, \tag{A48}$$

and substituting the noise model for the variance of the protein number gives:

$$k = k_0 - \tfrac{1}{2}|k''|\mu_p^2(\tfrac{\ln 2}{\mu_m} + C_0) - k_0\Lambda\mu_m, \tag{A49}$$

where C_0 is the noise floor, and we have assumed the mean protein number is optimal (μ_p). If we maximize the growth rate with respect to μ_m, we get the following condition on the optimal message number:

$$\hat{\mu}_m^2 = \tfrac{1}{2}\tfrac{|k''|}{k_0}\mu_p^2\tfrac{\ln 2}{\Lambda}, \tag{A50}$$

which depends on the unknown curvature k''. To make global predictions about how transcription and translation are related, some added assumptions are necessary to describe how k'' scales with protein abundance.

To illustrate how this expression does not make explicit global predictions, let's consider a number of plau-

sible possibilities. First, we will assume that k'' is independent of μ_p and on average all proteins are equally sensitive to changes in protein number. In this case, we find:

$$\mu_p \propto \hat{\mu}_m\sqrt{\Lambda}, \quad \text{(A51)}$$
$$\hat{\varepsilon} \propto \sqrt{\Lambda}, \quad \text{(A52)}$$

implying a constant translation efficiency which is inversely proportional to the square root of the relative load.

Alternatively, we can assume that $k'' \propto \mu_p^{-2}$ and, on average, the cell is equally sensitive to changes in the relative number of proteins (*i.e.* $\Delta p/\mu_p$), regardless of expression level. In this case,

$$\hat{\mu}_m \propto 1/\sqrt{\Lambda}, \quad \text{(A53)}$$
$$\hat{\varepsilon} \propto \mu_p\sqrt{\Lambda}, \quad \text{(A54)}$$

implying a constant message number, irrespective of expression level, and a translation efficiency that is proportional to expression level.

Finally, we will assume that $k'' \propto \mu_p^{-1}$, which is the intermediate case. Here:

$$\mu_p \propto \hat{\mu}_m^2\Lambda, \quad \text{(A55)}$$
$$\hat{\varepsilon} \propto \hat{\mu}_m\Lambda, \quad \text{(A56)}$$

implying that translation efficiency should increase with message number, analogous to our prediction. It appears that Hausser *et al.* implicitly also favor this model, since they define their sensitivity parameter to include a power of protein number μ_p. They justify this assumption by arguing that since $\sigma_p^2 \propto \mu_p$, it makes sense to define the *sensitivity to noise* to include a factor of μ_p [10]. At best, this is somewhat fuzzy logic since, as we demonstrate in the paper, Eq. A56 implies that the protein variance does not scale $\sigma_p^2 \propto \mu_p$!

The authors also propose a lower limit on the translation-transcription ratio; however, their limit is dependent on the noise floor, which only affects genes with the highest transcription rates in eukaryotic cells. The implementation of a more appropriate estimate of the noise, relevant for the vast majority of genes, does not lead to the same limit.

14. Methods: Analysis of translational limits & gene-specific load analysis

To explore the consequences of a protein-specific load, we can modify the metabolic load term in the growth rate equation (Eq. A18):

$$E\mu_m \to \lambda_p E\mu_m, \quad \text{(A57)}$$

which includes an additional parameter: the protein cost λ_p, which is 1 if the fitness cost is equal to the metabolic load and greater than one if the cost is higher. We will also treat the metabolic load per message λ as a gene-specific parameter in this section only. The optimization can be repeated for this augmented model.

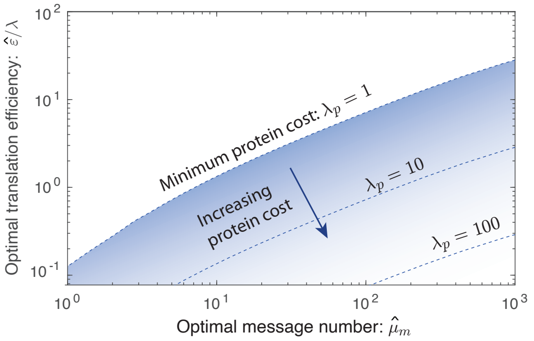

FIG. S8. **Increased protein cost decreases optimal translation efficiency.** A protein cost of $\lambda_p = 1$ corresponds to the metabolic cost of protein synthesis only, and is the minimum protein cost. For larger protein costs, the optimal translation efficiency is lower. As a result, the $\lambda_p = 1$ curve represents an upper bound of the optimal translation efficiency.

To analyze the affect of increased protein load, we modify Eq. A18:

$$\ln \tfrac{k}{k_0} = -(\Lambda + \lambda_p E)\mu_m - \tfrac{1}{\ln 2}\gamma\left(\tfrac{\mu_m}{\ln 2}, \tfrac{\phi_p}{E\ln 2}\right), \quad \text{(A58)}$$

to contain the supplemental load factor λ_p which is unity if the only protein load is metabolic and $\lambda_p > 1$ if there is additional load (*e.g.* toxicity). The optimization conditions (Eqs. A19 and A20) become:

$$0 = -\tfrac{\lambda + \lambda_p\hat{\varepsilon}}{N_0} - \tfrac{1}{(\ln 2)^2}\gamma_{,1}\left(\tfrac{\hat{\mu}_m}{\ln 2}, \tfrac{n_p}{\hat{\varepsilon}\ln 2}\right), \quad \text{(A59)}$$
$$0 = -\tfrac{\lambda_p\hat{\mu}_m}{N_0} + \tfrac{n_p}{\hat{\varepsilon}^2(\ln 2)^2}\gamma_{,2}\left(\tfrac{\hat{\mu}_m}{\ln 2}, \tfrac{n_p}{\hat{\varepsilon}\ln 2}\right). \quad \text{(A60)}$$

Using the same algebraic approach as before, we can derive the same optimal overabundance and load equations:

$$\Lambda \ln 2 = -\partial_{\hat{\mu}_m}\gamma\left(\frac{\hat{\mu}_m}{\ln 2}, \frac{\hat{\mu}_m}{\hat{o}\ln 2}\right), \quad \text{(A61)}$$
$$\hat{R} = \tfrac{1}{\Lambda \ln 2} p_\Gamma\left(\hat{\mu}_m \middle| \tfrac{\hat{\mu}_m}{\ln 2}, \hat{o}\ln 2\right); \quad \text{(A62)}$$

however, the relation between the load and the translation efficiency now has an extra factor: λ_p:

$$R = \frac{\lambda_p \varepsilon \mu_m}{\lambda \mu_m} = \frac{\lambda_p \varepsilon}{\lambda}, \quad \text{(A63)}$$

representing the modified total load ratio.

15. Results: Increased protein-specific cost reduces the optimal translation efficiency.

The relation between the overabundance and message number is unchanged (Eq. A35). This result can

be rationalized in the following way: The optimal overabundance is determined by the noise which is determined by message number only. This relation is unaffected by the added parameter λ_p. However, the optimal translation efficiency is affected:

$$\hat{\varepsilon} = \frac{\lambda}{\lambda_p}\hat{R}, \tag{A64}$$

where $\hat{R}$ is the optimal load ratio, defined by Eq. A38. The optimal curves are shown in Fig. S8.

How do these added considerations affect the RLTO predictions? First, we consider message and protein length. What are the optimal translation efficiencies for two proteins, one ten times the length of the other, at fixed protein number? In this case, we will assume that both the transcriptional cost (λ) as well as the translational cost (λ_p) increase tenfold. These increases cancel, resulting in the same optimal translation efficiency since it is only the relative cost of transcription to translation that is determinative of the translation efficiency.

Now consider a tenfold protein-specific increase in protein cost at fixed message cost and fixed protein number. The message number and translation efficiency would change by compensatory factors of 10:

$$\hat{\mu}_m \rightarrow 10 \cdot \hat{\mu}_m, \tag{A65}$$

$$\hat{\varepsilon}_m \rightarrow \frac{1}{10} \cdot \hat{\varepsilon}_m, \tag{A66}$$

to maintain the protein number.

Returning to our original motivation, we can understand how genes with a higher protein-to-message cost migrate downwards and rightwards off the optimal $\lambda_p = 1$ curve, predicting a cloud versus a narrow strip in proteome fraction measurements shown in Fig. S7. If the relative load Λ were directly measured, we would expect the predicted optimal translation efficiency curve for $\lambda_p = 1$ to lie at the top edge of the observed data cloud rather than the bisecting it. This bisection is the consequence of fitting an effective relative load parameter to the abundance data in the unaugmented RLTO model.

16. Discussion: Translation limits in *E. coli*

A critical assumption in the RLTO model to this point has been that the optimal central dogma parameters are realizable in the cell; however, translation can be limited by a number of different mechanisms. The superior performance of the constant- over the optimal-translation-efficiency model in *E. coli* (Fig. S7D) suggests that this assumption may not be satisfied for bacteria. How do translation limits affect the model phenomenology?

When considering possible limits on translation, there are two natural mechanisms: (i) ribosome-number limit, where the number of ribosomes in the cell limits translation and (ii) a ribosome-per-message limit, where the number of ribosomes per message is limiting. Assuming the ribosome-number-limit mechanism, the original unconstrained optimization problem can be recast as a constrained optimization problem where the protein cost λ_p is reinterpreted as a Lagrange multiplier to constrain the number of proteins translated (*e.g.* [11]). In spite of this reformulation, we would still predict the same functional form for the coupling between the optimal translation efficiency and message number. (*I.e.* it is still optimal to have a higher translation efficiency for highly-expressed genes even if the total number of proteins is fixed.) Therefore, the ribosome-number-limit mechanism cannot be the rationale for the constant translation efficiency observed in *E. coli*.

Assuming the ribosome-per-message-limit mechanism, we limit the translation efficiency to a restricted range of values. If the unconstrained optimum lies above this range, the optimum is at the maximum limiting value. If the unconstrained optima for all genes lie above the realizable range, the model predicts a translation efficiency uncoupled from the message number, as observed. These predictions are consistent with the observed central dogma regulatory program in *E. coli*. In added support of this hypothesis, Hausser *et al.* have argued that *E. coli* translates close to just such a ribosome-per-message limit as a consequence of the finite ribosome complex footprint on a message [10].

17. Methods: Estimate of the message cost and metabolic load

We can estimate the message cost λ from the known total protein number for yeast and mammalian cells. (For *E. coli* this estimate is not possible since the protein cost in not determinative of the translation efficiency.)

The optimal translation efficiency is (Eq. A23):

$$\hat{\varepsilon} = \frac{\lambda}{\Lambda \ln 2} p_\Gamma(\hat{\mu}_m | \tfrac{\hat{\mu}_m}{\ln 2}, \hat{o}\ln 2), \tag{A67}$$

and therefore the optimal protein number is:

$$\hat{\mu}_p = \hat{\mu}_m \hat{\varepsilon} = \frac{\lambda}{\Lambda \ln 2}\hat{\mu}_m p_\Gamma(\hat{\mu}_m | \tfrac{\hat{\mu}_m}{\ln 2}, \hat{o}\ln 2). \tag{A68}$$

We define the normalization constant A:

$$A = \textstyle\sum_i [\hat{\mu}_m \cdot \frac{1}{\Lambda \ln 2} p_\Gamma(\hat{\mu}_m | \frac{\hat{\mu}_m}{\ln 2}, \hat{o}\ln 2)]_i, \tag{A69}$$

where the index i runs over all genes. Now, by summing Eq. A68, over all genes, we derive an expression for the total protein number $N_p^{\rm tot}$ in terms of the message cost λ and the normalization constant A:

$$N_p^{\rm tot} = \lambda A. \tag{A70}$$

Solving for the protein cost results in the estimate:

$$\hat{\lambda} = \frac{N_p^{\rm tot}}{A}. \tag{A71}$$

This message cost estimate $\hat{\lambda}$ can then be plugged into the metabolic load definition (Eq. A17) to estimate the

multiplicity:

$$\hat{N}_0 \equiv L_0 + \hat{\lambda} N_m^{\text{tot}} + N_p^{\text{tot}}, \tag{A72}$$

where we have ignored the non-protein and non-message contributions to the load ($L_0 = 0$).

a. Detailed protocol

We first estimate the message numbers, as described in Sec. C 2 c, from data. For each gene i, we set the optimal message number equal to the observed message number and then compute the optimal overabundance from the message number using Eq. A26. (Since the result is independent of the assumed Λ value, we set an arbitrary initial value of $\Lambda = 10^{-5}$.) We then use these single gene optimal message number and overabundances to compute A using Eq. A69. In Eqs. A71 and A72, we use the N_p^{tot} from Tab. S2. N_m^{tot} is computed by summing the estimated message numbers.

b. Estimate the message cost and metabolic load in yeast

In yeast, the estimates are:

$$A = 4.8 \times 10^5, \tag{A73}$$
$$\hat{\lambda} = 1.0 \times 10^2, \tag{A74}$$
$$\hat{N}_0 = 6.2 \times 10^7, \tag{A75}$$
$$\hat{\Lambda} = 1.6 \times 10^{-6}, \tag{A76}$$

where the data sources are described in detail in Sec. C 1 b.

c. Estimate the message cost and metabolic load in human cells

In human cells, the estimates are:

$$A = 4.3 \times 10^6, \tag{A77}$$
$$\hat{\lambda} = 7.1 \times 10^2, \tag{A78}$$
$$\hat{N}_0 = 2.4 \times 10^9, \tag{A79}$$
$$\hat{\Lambda} = 2.9 \times 10^{-7}, \tag{A80}$$

where the data sources are described in detail in Sec. C 1 c.

d. Estimate of the modified relative load in bacterial cells

In bacterial cells, we will assume a constant translation efficiency model. We therefore use the modified relative load formula (Eq. A29) to estimate Λ'. We will assume that the load is dominated by proteins and messages:

$$N_0 = \sum_i (\lambda + \varepsilon)\mu_m = (\lambda + \varepsilon) N_m, \tag{A81}$$

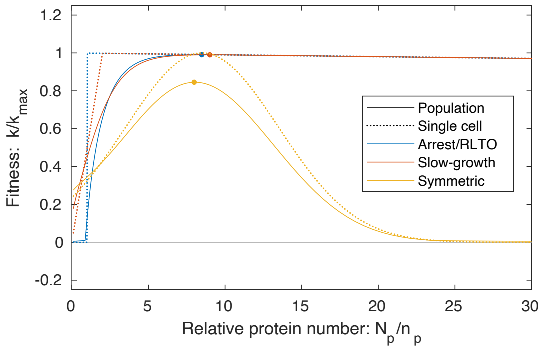

FIG. S9. **Exploring the mathematical mechanism of overabundance.** Single-cell and population growth rate are compared for three different models: arrest (RLTO), slow-growth, and symmetric models. In the arrest model (RLTO), the growth rate goes to zero below threshold protein level n_p. In the slow-growth model, the growth rate transitions continuously to zero as the N_p is depleted below n_p. In both the arrest and slow-growth models, there is a small negative slope above the threshold corresponding to the metabolic load. In the symmetric model, the fitness cost is symmetric about the optimum. Both the threshold-like and slow-growth models are optimized at mean expression levels μ_p far exceeding the threshold level n_p. This is a consequence of the highly-asymmetric dependence of the fitness on protein number N_p. This leads to the phenomenon of protein overabundance. In contrast, the symmetric model is optimized in close proximity to its single-cell optimum.

where N_m is the total number of messages. We can then solve this equation for Λ':

$$\hat{\Lambda}' = \tfrac{\lambda+\varepsilon}{N_0} = \tfrac{1}{N_m} \approx 10^{-5}, \tag{A82}$$

based on the total message number estimate for *E. coli*. (See Tab. S2.)

Appendix B: Model robustness & exploring alternatives to RLTO

In this section, we investigate the phenomenology of three different single-cell growth rate functions to determine what model features result in overabundance. We consider an *arrest model* (the RLTO model), a *slow-growth model*, and a *symmetric model*.

1. Methods: Defining alternative models

In each case, we will assume that the protein number is described by a gamma distribution:

$$N_p \sim \Gamma(\tfrac{\mu_m}{\ln 2}, \varepsilon \ln 2). \tag{B1}$$

We will assume the cell-cycle duration T is determined by this stochastic protein number N_p and then compute the population growth rate using Eq. A7 for a range of different message numbers μ_m. In each case, $\tau_0 = 1/N_0$, $N_0 = 10^5$, $\varepsilon = 30$, $n_p = \varepsilon \ln 2$. The mean expression level is $\mu_p = \mu_m \varepsilon$.

a. *Model 1: Arrest (RLTO) model*

The arrest (RLTO) model has cell cycle duration:

$$T = \tau_0 \begin{cases} \infty, & N_p < n_p \\ N_0 + N_p, & N_p > n_p \end{cases}, \tag{B2}$$

where protein expression below threshold n_p results in growth arrest.

b. *Model 2: Slow-Growth model*

In the slow-growth model, we imagine two processes: (i) checkpoint process X and (ii) other processes. The cell will divide after whichever process finishes last. Other processes will finish after time predicted by the metabolic load, identical to the threshold model defined above. However, we model checkpoint process X as the completion of a fixed amount of activity in an irreversible process. We will therefore assume it will take a time inversely proportional to the amount of enzyme X (N_p). The amount of activity is set by effective threshold n_p:

$$T = \tau_0 \max\{N_0 + N_p, \tfrac{2n_p N_0}{N_p}\} \tag{B3}$$

such that n_p defines the level of protein required to make the growth rate half the metabolic limit.

Unlike the arrest model, cell growth slows but does not stop for $N_p < n_p$. This model will test whether the results of the RLTO model are an artifact of the assumed arrest-based slow growth.

c. *Model 3: Symmetric model*

For the symmmetric model, we choose the model parameters such that the single-cell optimum was close to the other models: $n_0 = 8.5$, $\sigma_n = 5$. The cell-cycle duration is:

$$T = \tau_0 N_0 \exp\left(\tfrac{(N_p - n_0)^2}{2\sigma_n^2}\right), \tag{B4}$$

such that the noise-free growth rate will be Gaussian is N_p.

2. Results: Overabundance is a robust prediction

The growth rates as a function of the mean expression level μ_p are shown in Fig. S9. The symmetric model has a population optimum in close proximity to its single-cell optimum, as we intuitively expect. However, both the arrest (RLTO) model and the slow-growth model have optima far above the threshold number n_p. We therefore conclude that it is fitness asymmetry rather than growth arrest that is responsible for the overabundance phenomenon.

Why doesn't growth arrest of a sub-population lead to a stronger effect than the same sub-population growing slowly? In Ref. [2], we showed that the population doubling time $\overline{T}$ can be understood as the exponential mean of the stochastic cell-cycle duration:

$$\overline{T} \equiv f^{-1}[\mathbb{E}_T f(T)], \tag{B5}$$

where $\mathbb{E}_T$ is the expectation over the stochastic duration T and $f(t) \equiv \exp(-kt)$, where $k = \overline{T}^{-1} \ln 2$ is the population growth rate. Due to the functional form of $f(t)$, any long cell cycles are exponentially suppressed in their contribution to the exponential mean. Therefore, low-probability extremely-long-duration cell cycles only contribute to the growth rate by reducing the fraction of growing cells.

Appendix C: Quantitation of central dogma parameters for one-message-rule

The RLTO model predicts the *one-message-rule* for the lower threshold on transcription for essential genes. In this section, we use transcriptome data from the literature to test this prediction. We first describe the sources of the data (Sec. C 1), how the estimates are computed (Sec. C 2), the results (Sec. C 3) and discussion (Sec. C 4).

1. Methods: Selection of central dogma parameter estimates

The estimates for central dogma model parameters come from two types of data: (i) quantitative measurement of cellular-scale parameters for each organism (total number of messages in the cell, cell cycle duration, *etc*) and (ii) genome-wide studies quantitative of mRNA and protein abundance.

For the cellular-scale central dogma parameters, we relied heavily on an online compilation of biological numbers: BioNumbers [22]. This resource provides a collection of curated quantitative estimates for biological numbers, as well as their original source. In the interest of conciseness, we have cited only the original source in the Tab. S2, although we are extremely grateful and supportive of the creators of the BioNumbers

Model organism	Growth condition	Doubling time:	Message lifetime:	Message recycling ratio:	Total number of messages /cell:	messages /cell-cycle:	proteins:	Average translation efficiency:	translation rate:
		T	$\tau_m = \gamma_m^{-1}$	$m = T/\tau_m$	$N_{m/c}^{\text{tot}}$	N_m^{tot}	N_p^{tot}	ε	β_p (h^{-1})
Escherichia coli (*E. coli*)	LB	30 min [12]	2.5 min [13]	12	7.8×10^3 [14]	9.4×10^4	3×10^6 [15]	22	530
	M9	90 min [12]	2.5 min [13]	36	2.4×10^3 [14]	8.6×10^4	3×10^6 [15]	24	580
Sacchromyces cerevisiae (Yeast–haploid)	YEPD	90 min [16]	22 min [17]	4	2.9×10^4 [18]	1×10^5	5×10^7 [19]	4×10^2	410
Mus musculus (Mammalian-mouse)	Tissue	27.5 h [9]	15 h [9]	1.8	1.7×10^5 [9]	3×10^5 [9]	3×10^9 [9]	1×10^4	660
Homo sapiens (Human)	Tissue	24 h [20]	14 h [21]	1.7	3.6×10^5 [16]	5×10^5	2×10^9 [15]	4×10^3	120

TABLE S2. **Central dogma parameters for three model organisms with detailed references.** Columns three through seven hold representative values for measured central-dogma parameters for the model organisms described in the paper. Each value is followed by a reference for its source.

website for helping us very efficiently identify consensus estimates for the parameters of the central dogma parameters.

For the selection of genome-wide studies on abundance, we used many of the same resources cited in BioNumbers as well as studies selected by a previous study of a quantitative analysis of the central dogma: Hausser *et al.* [10].

a. *E. coli data*

Message lifetimes: The message lifetimes (and median lifetime) were taken from a recent transcriptome-wide study by Chen *et al.* [13]. These investigators measured the lifetime in both rapid (LB) and slow growth (M9).

Noise: Taniguchi *et al.* have performed a beautiful simultaneous study of the proteome and transcriptome with single-molecule sensitivity [4]. Although we use the noise analysis data from this study for our supplemental analysis of *E. coli* noise, it is not the source for our *E. coli* transcriptome data due to the extremely slow growth of the cells in this study (150 minute doubling time), which is not consistent with the growth conditions for the other sources of data.

mRNA abundance: Instead, we used data from the more recent Bartholomaus *et al.* study [14], which characterizes the transcriptome in both rapid (LB) and slow growth (M9).

Total cellular message number. This study was chosen since it was the source of the BioNumbers estimates of cellular message number in *E. coli* (BNID 112795 [22]).

Doubling time: The source of the doubling times for rapid (LB) and slow (M9) growth of *E. coli* comes from Bernstein [12].

Essential gene classification. The classification of essential genes in yeast comes from the construction of the Keio knockout collection from Baba *et al.* [23].

Protein number. The total protein number in *E. coli* came from Milo's recent review of this subject [15].

b. *Yeast data*

Message lifetimes: The message lifetimes (and median lifetime) were taken from Chia *et al.* [17].

Noise: The noise data was taken from the Newman *et al.* study, which used flow cytometry of a library of fluorescent fusions to characterize protein abundance with single-cell resolution [24].

mRNA abundance: The transcriptome data comes from the very recent Blevins *et al.* study [25].

Total cellular message number. There are a wide-range of estimates for the total cellular message number in yeast: 1.5×10^4 [26] (BNID 104312 [22]), 1.2×10^4 [27] (BNID 102988 [22]), 6.0×10^4 [28] (BNID 103023 [22]), 2.6×10^4 [18] (BNID 106763 [22]) and 3.0×10^4 [29]. We used the compromise value of 2.9×10^4.

Doubling time: The doubling time was taken from [16].

Protein number. The total protein number in yeast comes from Futcher *et al.* [19].

Essential gene classification. The classification of essential genes in yeast comes from van Leeuwen *et al.* [30].

Proteome abundance data: The proteome abundance data came from two sources: flow cytometry of fluorescent fusions from Newman *et al.* [24] as well as mass-spec data from de Godoy *et al.* [31].

c. Human data

Message lifetimes: The message lifetimes (and median lifetime) were taken from Yang *et al.* [21] who reported a median half life of 10 h which corresponds to a lifetime of 14 h.

mRNA abundance: The transcriptome data comes from the data compiled by the Human Protein Atlas [32], which we averaged over tissue types.

Total cellular message number. The total cellular message number in human comes from Velculescu *et al.* [33] (BNID 104330 [22]).

Doubling time: The doubling time was taken from [20].

Protein number. The total protein number in human came from Milo's recent review of this subject [15].

Essential gene classification. The classification of essential genes in human comes from Wang *et al.* [34].

2. Methods: Quantitative estimates of central dogma parameters

a. Estimating the cellular message number: $\mu_{m/c}$

For each model organism (and condition), we found a consensus estimate from the literature for the total number of mRNA messages per cell $N_{m/c}^{\rm tot}$. This number and its source are provided in Tab. S2. To estimate the number of messages corresponding to gene i, we re-scaled the un-normalized abundance level r_i:

$$N_{m/c,i} = N_{m/c}^{\rm tot}\frac{r_i}{\sum_j r_j}, \tag{C1}$$

where the sum over gene index j runs over all genes.

b. Estimating the transcription rate: β_m

To estimate the transcription rate for gene i, we start from the estimated cellular message number $N_{m/c,i}$ and use the telegraph model prediction for the cellular message number:

$$N_{m/c,i} = \beta_{m,i}/\gamma_{m,i}, \tag{C2}$$

where $\gamma_{m,i}$ is the message decay rate. Since gene-to-gene variation in message number is dominated by the transcription rate (*e.g* [13]), we estimate the decay rate as the inverse gene-median message life time:

$$\gamma_{m,i} = \tau_m^{-1}, \tag{C3}$$

for which a consensus value was found from the literature. This number and its source are provided in Tab. S2. We then estimate the gene-specific transcription rate:

$$\beta_{m,i} = N_{m/c,i}/\tau_m. \tag{C4}$$

c. Estimating the message number: μ_m

To estimate the message number of gene i, we use the predicted value from the telegraph model:

$$N_{m,i} = T\beta_{m,i} = \frac{T}{\tau_m}N_{m/c,i}, \tag{C5}$$

where T is the doubling time and $N_{m/c,i}$ is the cellular message number (Eq. C1).

3. Results: Histograms of central dogma transcriptional statistics

We generated histograms for each of the three transcriptional statistics: transcription rate β_m, cellular message number $\mu_{m/c}$, and message number μ_m. The histograms for transcription rate and cellular message number do not show a consistent lower limit (as predicted) and are shown in Fig. S10; however, the histogram for message number does show a consistent lower bound for the three model organisms and is shown in Fig. S10B.

4. Discussion: *E. coli* essential genes below the one-message-rule threshold

Since our own preferred model system is *E. coli*, we focus here. Our essential gene classification was based on the construction of the Keio knockout library [23]. By this classification, 10 essential genes were below threshold. (See Tab. S3.) Our first step was to determine what fraction of these genes were also classified as essential using transposon-based mutagenesis [35, 36]. Of the 10 initial candidates, only one gene, *ymfK*, was consistently classified as an essential gene in all three studies, and we estimate that its message number is just below the threshold ($\mu_m = 0.4$). *ymfK* is located in the lambdoid prophage element e14 and is annotated as a CI-like repressor which regulates lysis-lysogeny decision [37]. In λ phase, the CI repressor represses lytic genes to maintain the lysogenic state. A conserved function for *ymfK* is consistent with it being classified as essential, since its regulation would prevent cell lysis. However, since *ymfK* is a prophage gene, not a host gene, it is not clear that its expression should optimize host fitness, potentially at the expense of phage fitness. In summary, closer inspection of below-threshold essential genes supports the threshold hypothesis.

Appendix D: Analysis of gene-expression noise

This section provides a detailed development of gene expression noise. Sec. D1 provides a self-contained development of the noise models developed by others

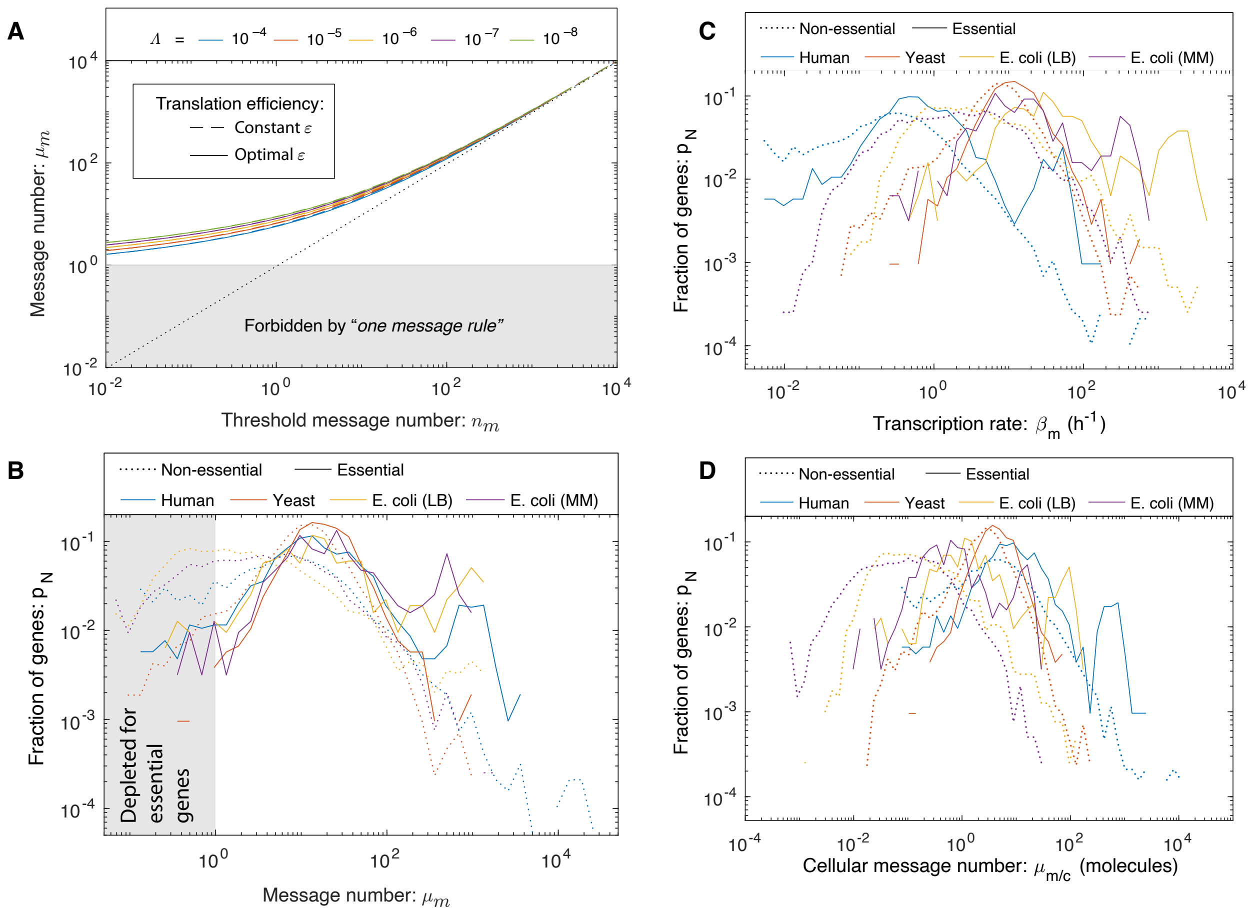

FIG. S10. **Panel A: One-message-rule for essential genes.** For highly transcription genes (high μ_m), little compensation for noise is required and the optimal message number tracks with the threshold message number n_m. However, as the threshold message number approaches one ($n_m \to 1$), the noise is comparable to the mean, and the optimal message number μ_m increases to compensate for the noise. As a result, a lower threshold of roughly one message per cell-cycle is required for essential genes. This threshold is predicted for both fixed (dashed) and optimized translation efficiency (solid). The threshold is weakly dependent on relative load Λ. **Panel B: A one message threshold is observed in three evolutionarily-divergent organisms.** As predicted by the RLTO model, essential, but not nonessential genes, are observed to be expressed above a one message per cell-cycle threshold. All organisms have roughly similar distributions of message number for essential genes, which are not observed for message numbers below a couple per cell cycle. **Panel C: The distribution of gene transcription rate.** The typical transcription rate varies by two orders-of-magnitude between organisms. **Panel D: The distribution of gene cellular message number.** There is also a two-order-of-magnitude variation between typical cellular message numbers. No consistent lower threshold is observed for either statistic.

which are the input to the RLTO model. Secs. D 2-D 7 describe the RLTO prediction of non-canonical noise scaling and the test of this model.

1. Methods: Detailed description of the noise model

a. Telegraph model: Stochastic kinetic model for central dogma.

The telegraph model for the central dogma describes multiple steps in the gene expression process: Transcription generates mRNA messages [38]. These messages are then translated to synthesize the protein gene products [38]. Both mRNA and protein are subject to degradation and dilution [39]. At the single cell level, each of these processes are stochastic. We will model these processes with the stochastic kinetic scheme [38]:

$$\begin{array}{ccccc} \text{DNA} & \xrightarrow{\beta_m} & \text{mRNA} & \xrightarrow{\beta_p} & \text{Protein} \\ & & \big\downarrow{\scriptstyle \gamma_m} & & \big\downarrow{\scriptstyle \gamma_p} \\ & & \emptyset & & \emptyset, \end{array} \tag{D1}$$

where β_m is the transcription rate (s^{-1}), β_p is the translation rate (s^{-1}), γ_m is the message degradation rate (s^{-1}), and γ_p is the protein effective degradation rate (s^{-1}). The message lifetime is $T_m \equiv \gamma_m^{-1}$. For most proteins in the context of rapid growth, dilution is the dominant

Gene name	Message number: μ_m	Annotated function from Ecocyc	Essential (E)/ Nonessential (N) Ref. [23], [35], [36]
alsK	0.3	The alsK gene encodes a D-allose kinase. Its role in the degradation of D-allose is unclear; AlsK is not required for utilization of a D-allose carbon source; this effect may be due to the presence of other ambiguous sugar kinases within *E. coli* K-12.	E, N, N
bcsB	0.4	BcsB is encoded in a predicted operon together with *bcsA*, *bcsZ* and *bcsC*. In other organisms, these genes are involved in cellulose biosynthesis, a characteristic of the rdar (red, dry and rough) morphotype. However, the K-12 laboratory strain of *E. coli* does not show a rdar morphotype and does not produce cellulose.	E, N, N
entD	0.4	AcpS is the founding member of a 4'-phosphopantetheinyl (P-pant) transferase protein family that includes *E. coli* EntD, *E. coli* o195 protein, and *Bacillus subtilis* Sfp; family members share two conserved motifs but relatively low sequence identity overall.	E, N, N
yafF	0.4	No information about this protein was found by a literature search conducted on April 19, 2017.	E,-, N
yagG	0.6	*yagGH* is predicted to be a member of the XylR regulon; its products may mediate transport (YagG) and hydrolysis (YagH) of xylooligosaccharides; putative XylR and CRP binding sites are identified upstream of *yagGH*.	E,-, N
yceQ	0.2	No information about this protein was found by a literature search conducted on July 12, 2017.	E, E, N
ydiL	0.2	No information about this protein was found by a literature search conducted on April 7, 2017.	E, N, N
yhhQ	0.4	YhhQ is an inner membrane protein implicated in the uptake of queuosine (Q) precursors - 7-cyano-7-deazaguanine (preQ0) and 7-aminomethyl-7-deazaguanine (*preQ1*) - for Q salvage. Q-modified tRNA is absent in Δ*queD* and Δ*queD* Δ*yhhQ* strains grown in minimal media with glycerol; Q-modified tRNA is detected when a Δ*queD* strain is grown in minimal media plus 10 nM *preQ0* or *preQ1* but is absent when a Δ*queD* Δ*yhhQ* strain is grown under these conditions. *yhhQ* expressed from a plasmid restores the presence of Q-modified tRNA in a Δ*queD* Δ*yhhQ* strain.	E,-, N
yibJ	0.3	No information about this protein was found by a literature search conducted on July 9, 2018.	E, N, N
ymfK	0.4	YmfK is a component of the relic lambdoid prophage e14 and is likely the SOS-sensitive repressor. It is similar to the P34 gene of the *Shigella flexneri* bacteriophage SfV and belongs to the LexA group of SOS-response transcriptional repressors.	E, E, E

TABLE S3. **Below-threshold essential genes identified in *E. coli*.** This table describes the message numbers and annotations for essential genes that we estimated to have expression below the threshold of one message per cell cycle. However, in the final column, we show classifications from three different studies. Only one of the identified genes, *ymfK*, was consistently defined as essential.

mechanism of protein depletion and therefore γ_p is approximately the growth rate [4, 40, 41]: $\gamma_p = T^{-1}\ln 2$, where T is the doubling time.

b. Statistical model for protein abundance.

To study the stochastic dynamics of gene expression, we used a stochastic Gillespie simulation [42, 43]. (See Sec. D 1 c.) In particular, we were interested in the explicit relation between the kinetic parameters $(\beta_m, \gamma_m, \beta_p, \gamma_p)$ and experimental observables.

Consistent with previous reports [44, 45], we find that the distribution of protein number per cell (at cell birth) was described by a gamma distribution: $N_p \sim \Gamma(a, \theta)$, where N_p is the protein number at cell birth and Γ is the gamma distribution which is parameterized by a scale parameter θ and a shape parameter a. (See Sec. D 1 d.)

The relation between the four kinetic parameters and these two statistical parameters has already been reported, and have clear biological interpretations [45]: The scale parameter:

$$\theta = \varepsilon \ln 2, \tag{D2}$$

is proportional to the translation efficiency:

$$\varepsilon \equiv \frac{\beta_p}{\gamma_m}, \tag{D3}$$

where β_p is the translation rate and γ_m is the message degradation rate. ε is understood as the mean number of proteins translated from each message transcribed. The shape parameter a can also be expressed in terms of the kinetic parameters [45]:

$$a = \frac{\beta_m}{\gamma_p}; \tag{D4}$$

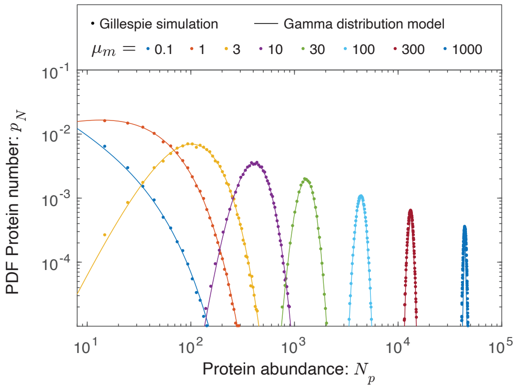

FIG. S11. **The protein abundance is approximately gamma distributed.** Protein abundance was modeled for eight different transcription rates using a Gillespie simulation, including the stochastic partitioning of the proteins between daughter cells at cell division. The range in abundance matches the observed range of expression levels in the cell. We observed that the simulated protein abundances were well fit by gamma distributions.

however, we will find it more convenient to express the scale parameter in terms of the cell-cycle message number:

$$\mu_m \equiv \beta_m T = a \ln 2, \tag{D5}$$

which can be interpreted as the mean number of messages transcribed per cell cycle. Forthwith, we will abbreviate this quantity *message number* in the interest of brevity.

c. Gillespie simulation of the telegraph model

Protein distributions of the telegraph model for *E. coli* were simulated with a Gillespie algorithm. Assuming the lifetime of the cell cycle ($T = 30$ min) [12], mRNA lifetime ($T_m = 2.5$ min) [13], and translation rate ($\beta_p \approx 500$ hr^{-1}), the protein distributions for several mean expression levels were numerically generated for exponential growth with 100,000 stochastic cell divisions, with protein partitioned at division following the binomial distribution.

The gamma distributions for each mean message number with scale and shape parameters determined by the corresponding translation efficiency and message number ($\theta = \varepsilon \ln 2$, $a = \frac{\mu_m}{\ln 2}$) as used for the Gillespie simulation were also plotted with the protein distributions. We observe an excellent match between these Gillespie simulations and the statistical noise model (*i.e.* gamma function) as shown in Fig. S11.

d. Gamma function and distribution conventions

There are a number of conflicting conventions for the gamma function and distribution arguments. We will use those defined on Wikipedia [46]. The gamma distributed random variable X will be written:

$$X \sim \Gamma(a, \theta), \tag{D6}$$

where a is the shape parameter and θ is the scale parameter. The PDF of the distribution is:

$$p_\Gamma(x|a,\theta) \equiv \tfrac{x^{a-1}}{\theta^a \Gamma(a)} e^{-x/\theta}, \tag{D7}$$

where $\Gamma(a)$ is the gamma function. The CDF is therefore:

$$\begin{aligned} P_\Gamma(x|a,\theta) &= \int_0^x \mathrm{d}x'\ p_\Gamma(x'|a,\theta), && \text{(D8)}\\ &= P_\Gamma(\tfrac{x}{\theta}|a,1), && \text{(D9)}\\ &= \int_0^{x/\theta} \mathrm{d}x'\ \tfrac{x^{a-1}}{\Gamma(a)} e^{-x}, && \text{(D10)}\\ &= \gamma(a, x/\theta), && \text{(D11)} \end{aligned}$$

where γ is the regularized incomplete gamma function.

2. Results: RLTO model predicts non-canonical noise scaling

The predicted scaling of the optimal translation efficiency with message number has many important implications, including on the global characteristics of noise. Based both on theoretical and experimental evidence, it is widely claimed that gene-expression noise should be inversely proportional to protein abundance [4, 47]:

$$\mathrm{CV}_p^2 \propto \mu_p^{-1}, \tag{D12}$$

for low-expression proteins, as observed in *E. coli* [4]; however, the more fundamental prediction is that the noise is inversely proportional to the message number:

$$\begin{aligned} \mu_p &= \mu_m \varepsilon, && \text{(D13)}\\ \mathrm{CV}_p^2 &= \tfrac{\ln 2}{\mu_m}. && \text{(D14)} \end{aligned}$$

In *E. coli*, the translation efficiency is roughly constant (*i.e.* $\hat{\mu}_p \propto \hat{\mu}_m$, Fig. S7D) and therefore Eq. D14 is consistent with the canonical noise model (Eq. D12). However, in eukaryotes, the translation efficiency grows with message number (*i.e.* $\hat{\mu}_p \propto \hat{\mu}_m^2$, Fig. S7BC). If we substitute this proportionality into Eq. D14, we predict the non-canonical noise scaling:

$$\mathrm{CV}_p^2 \propto \mu_p^{-1/2}, \tag{D15}$$

for eukaryotic cells.

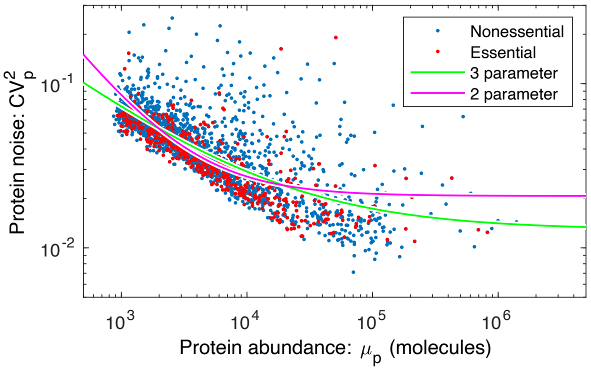

FIG. S12. **Yeast noise fit against canonical noise model, with a noise floor.** Yeast noise data fit with the 2- (null hypothesis with μ_p^{-1} dependence) and 3- parameter (μ_p^a) models. The two-parameter model corresponds to the canonical noise model (Eq. D12) and fails to quantitatively fit the data.

3. Methods: Analysis of gene expression noise

The quantitative model for gene expression noise includes multiple contributions:

$$\mathrm{CV}_p^2 \approx \tfrac{1}{\mu_p} + \tfrac{\ln 2}{\mu_m} + c_0, \tag{D16}$$

where the first term can be understood to represent the Poisson noise from translation, the second term the Poisson noise from transcription, and the last term, c_0, is called the *noise floor* and is believed to be caused by the cell-to-cell variation in metabolites, ribosomes, and polymerases *etc.* [48, 49].

In the main text of the paper, we have ignored the role of the noise floor in the analysis of noise in yeast. Unlike *E. coli*, where the noise floor is high ($\mathrm{CV}_p^2 = 0.1$) and is determinative of the noise associated with almost all essential genes [4, 48, 49], in yeast the noise floor is much lower ($\mathrm{CV}_p^2 = 0.01$) and therefore affects only genes with the highest expression.

In this section, we will consider models that include the noise floor, since its presence can make the noise scaling more difficult to interpret. To determine if the scaling of the noise is consistent with the canonical assumption that the noise is proportional to μ_p^{-1} for low expression, we will consider two competing empirical models for the noise (Fig. S12). In the null hypothesis, we will consider a model:

$$\eta_0(\mu_p; b, c) = \tfrac{b}{\mu_p} + c, \tag{D17}$$

and an alternative hypothesis with an extra exponent parameter a:

$$\eta_1(\mu_p; a, b, c) = \tfrac{b}{\mu_p^a} + c. \tag{D18}$$

We will assume that CV_p^2 is normally distributed about η with unknown variance σ_η^2.

In this context, a maximum likelihood analysis is equivalent to least-squares analysis. Let the sum of the squares be defined:

$$S_I(\boldsymbol{\theta}) \equiv \sum_i [\mathrm{CV}_{p,i}^2 - \eta_I(\mu_{p,i}; \boldsymbol{\theta})]^2, \tag{D19}$$

for model I where $\boldsymbol{\theta}$ represents the parameter vector. The maximum likelihood parameters are

$$\hat{\boldsymbol{\theta}} = \arg\max_{\boldsymbol{\theta}} S_I(\boldsymbol{\theta}), \tag{D20}$$

with residual norm:

$$\hat{S}_I = S_I(\hat{\boldsymbol{\theta}}). \tag{D21}$$

To test the null hypothesis, we will use the canonical likelihood ratio test with the test statistic:

$$Z \equiv 2 \ln \frac{q_1}{q_0}, \tag{D22}$$

where q_0 and q_1 are the likelihoods of the null and alternative hypotheses, respectively. Wilks' theorem states that Z has a chi-squared distribution of dimension equal to the difference of the dimension of the alternative and null hypotheses ($3 - 2 = 1$).

a. *Hypothesis test I*

In our first analysis, we will estimate the variance directly. We computed the mean-squared difference for successive CV_p^2 values, sorted by mean protein number μ_p. The variance estimator is

$$\hat{\sigma}_\eta^2 = \tfrac{1}{2} \left\langle (\mathrm{CV}_{p,i}^2 - \mathrm{CV}_{p,i+1}^2)^2 \right\rangle_i = 6.3 \times 10^{-4}, \tag{D23}$$

where the brackets represent a standard empirical average over gene i for the μ_p-ordered gene CV_p^2 values. The test statistic can now be expressed in terms of the residual norms:

$$\begin{aligned} Z &= (\hat{S}_1 - \hat{S}_2)/\hat{\sigma}_\eta^2, & \text{(D24)} \\ &= 3.3 \times 10^4, & \text{(D25)} \end{aligned}$$

which corresponds to a p-value far below machine precision. We can therefore reject the null hypothesis.

b. *Hypothesis test II*

In a more conservative approach, we can use maximum likelihood estimation to estimate the variance of each model independently as a model parameter. In this case, the test statistic can again be expressed in terms of the residual norms:

$$\begin{aligned} Z &= N \ln \tfrac{\hat{S}_1}{\hat{S}_2}, & \text{(D26)} \\ &= 1.6 \times 10^2, & \text{(D27)} \end{aligned}$$

where N is the number of data points. (Details of derivation are in Sec. D 3 d.) In this case, the p-value can be computed assuming the Wilks' theorem (*i.e.* the chi-squared test):

$$p = 6 \times 10^{-36}, \tag{D28}$$

again, strongly rejecting the null hypothesis.

c. Maximum likelihood estimates of the parameters

In the alternative hypothesis, the maximum likelihood estimate (MLE) of the empirical noise model (Eq. D18) parameters are (Fig. S12):

$$a = 0.57 \pm 0.02, \tag{D29}$$
$$b = 3.0 \pm 0.5, \tag{D30}$$
$$c = 0.013 \pm 0.001, \tag{D31}$$

where the parameter uncertainty has been estimated using the Fisher Information in the usual way using the MLE estimate of the variance [50, 51].

d. Details: Statistical details MLE estimate of the variance

The minus-log-likelihood for the normal model I is:

$$h_I(\hat{\boldsymbol{\theta}}, \sigma^2) = \tfrac{N}{2} \ln 2\pi\sigma^2 + \tfrac{1}{2\sigma^2}\hat{S}_I, \tag{D32}$$

where $\hat{S}_I$ is the least-square residual. We then minimize h_I with respect to the variance σ^2:

$$\partial_{\sigma^2} h|_{\hat{\sigma}^2} = 0, \tag{D33}$$

to solve for the MLE $\hat{\sigma}^2$:

$$\hat{\sigma}^2 = \tfrac{1}{N}\hat{S}_I. \tag{D34}$$

Next we evaluate h at the variance estimator:

$$h_I(\hat{\boldsymbol{\theta}}, \hat{\sigma}^2) = \tfrac{N}{2}\left[\ln 2\pi \tfrac{\hat{S}_I}{N} + 1\right]. \tag{D35}$$

The test statistics can be written in terms of the h's:

$$Z = 2h_0(\hat{\boldsymbol{\theta}}, \hat{\sigma}^2) - 2h_1(\hat{\boldsymbol{\theta}}, \hat{\sigma}^2), \tag{D36}$$
$$= N \ln \tfrac{\hat{S}_0}{\hat{S}_1}, \tag{D37}$$

which can be evaluated directly in terms of the residual norms for the null and alternative hypotheses.

4. Results: Non-canonical noise scaling is observed in yeast.

To test the RLTO model predictions for noise scaling, we reanalyze the dataset collected by Newman *et al.*, who performed a single-cell proteomic analysis of yeast by measuring the abundance of fluorescent fusions by flow cytometry [24]. Since the competing models (Eqs. D12 and D15) make different scaling predictions, we first apply a statistical test to determine whether the observed scaling is consistent with the canonical model (Eq. D12). We consider the null hypothesis of the canonical model (Eq. D17) and the alternative hypothesis with an unknown scaling exponent (Eq. D18). To test the models, we perform a null hypothesis test. (A detailed description of the statistical analysis, which include the contribution of the noise floor, is given in the Sec. D 1 d.) We reject the null hypothesis with a p-value of $p = 6 \times 10^{-36}$. The observed scaling exponent is $\hat{a} = -0.57 \pm 0.02$, which is close to our predicted estimated exponent from the RLTO model ($-\frac{1}{2}$).

5. Methods: Parameter-free prediction of noise from protein-message relation.

By combining the noise model (Eq. D14) with a protein-message abundance relation, the relation between protein abundance and noise can be predicted without additional fitting parameters. (We call this prediction *parameter-free* since, although a parameter is fit when determining the protein-message abundance, once this relation has been established, no new parameters are fit in order to predict the noise.) To test this prediction, we will compare three competing models: (i) the RLTO model, (ii) an empirical protein-message abundance model, and (iii) the constant-translation-efficiency model.

a. Estimating protein number (μ_p) for the noise analysis

The protein abundance data for yeast grown in YEPD media and measured with flow cytometry fluorescence [24] were given in arbitrary units (AU). In order to convert from AU to protein number, the fluorescence values were rescaled by comparing with mass-spectrometry protein abundance data for yeast grown in YNB media [31]. Since the protein abundance from mass-spectrometry was given in terms of intensity, the intensity values were first rescaled by the total number of proteins in yeast, 5×10^7. (See Sec. C 1 b.) The mass-spectrometry protein data was thresholded at 10 proteins, based on the assumption that the noise of the data for 10 and fewer proteins makes the data unreliable. Next, the log of the fluorescence protein abundance in AU as a function of the log of thresholded mass-spectrometry protein abundance was fit as a linear function with an assumed slope of 1 to find the offset, 3.9, (Fig. S13) which corresponds to a multiplicative scaling factor. We then used that offset value to rescale the fluorescence data from AU to protein number. We also compared to yeast grown in SD media [24] and found a

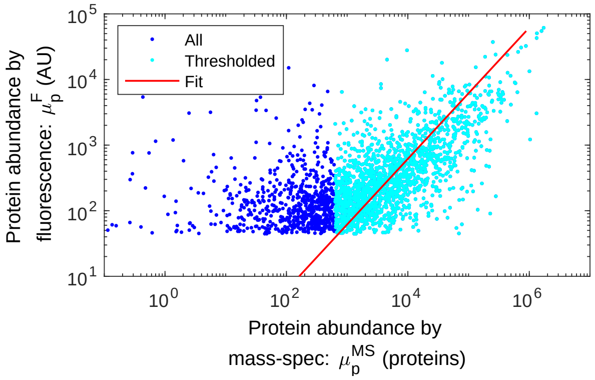

FIG. S13. **Fit to rescale fluorescence intensity to protein number.** Protein abundance from flow cytometry fluorescence [24] as a function of mass-spectrometry scaled abundance [31]. The mass-spectrometry data was thresholded at 10 proteins, and then a linear fit was performed to find the multiplicative offset of 3.9, which was used to convert protein fluorescence AU to number.

similar offset result.

b. *Empirical models for yeast gene expression*

To generate the empirical model for protein number as a function of message number, we used protein abundance data from Newman *et al.* [24], re-scaled to estimate protein number (Sec. D 5 a) and transcriptome data from Lahtvee *et al.* [52], re-scaled to estimate message number (Sec. C 2 c).

c. *Empirical model for protein number*

We initially fit the empirical model for protein number,

$$\mu_p = C_0 \mu_m^{\alpha_0}, \tag{D38}$$

to the data using a standard least-squares approach; however, the algorithm led to a very poor fit since it does not account for uncertainty in both independent and dependent variables. We therefore used an alternative approach [53], which assumes comparable error in both variables. The model parameters are:

$$\alpha_0 = 2.1 \pm 0.04, \tag{D39}$$
$$C_0 = 8.0 \pm 1.0, \tag{D40}$$

where the uncertainties are the estimated standard errors. The result of the empirical model fit is shown in Fig. S14A, along with the constant-translation-efficiency model, and the RLTO model.

d. *Empirical model for message number*

For the prediction of the coefficient of variation, it is useful to invert Eq. D38 to generate a model for message number as a function of protein number:

$$\mu_m = C_0^{-1/\alpha_0} \mu_p^{1/\alpha 0}, \tag{D41}$$
$$= C_1 \mu_p^{\alpha_1}, \tag{D42}$$

where the last line defines two new parameters: a coefficient C_1 and an exponent α_1. The resulting parameters and uncertainties are:

$$\alpha_1 \equiv 1/\alpha_0, \tag{D43}$$
$$= 0.48 \pm 0.01, \tag{D44}$$
$$C_1 \equiv C_0^{-1/\alpha_0}, \tag{D45}$$
$$= 0.37 \pm 0.02, \tag{D46}$$

where the uncertainties are the estimated standard errors.

e. *Empirical model for translation efficiency*

To generate an empirical model for translation efficiency, we started from the empirical model for protein number (Eq. D38), and then use Eq. D13 to relate protein number, message number, and translation efficiency:

$$\varepsilon = \frac{\mu_p}{\mu_m}, \tag{D47}$$
$$= C_0 \mu_m^{\alpha_0 - 1}, \tag{D48}$$
$$= C_2 \mu_m^{\alpha_2}, \tag{D49}$$

where the last line defines two new parameters: a coefficient C_2 and an exponent α_2. The resulting parameters and uncertainties are:

$$\alpha_2 = \alpha_0 - 1, \tag{D50}$$
$$= 1.07 \pm 0.04, \tag{D51}$$
$$C_2 = C_0, \tag{D52}$$
$$= 8.0 \pm 1.0, \tag{D53}$$

where the uncertainties are the estimated standard errors.

f. *Empirical model for the coefficient of variation*

To generate an empirical model for the coefficient of variation, we started from the empirical model for message number (Eq. D42), and then substitute this into the statistical model prediction for CV_p^2 (Eq. D14):

$$\mathrm{CV}_p^2 = \frac{\ln 2}{\mu_m}, \tag{D54}$$
$$= C_0^{1/\alpha_0} \ln 2 \cdot \mu_p^{-1/\alpha_0}, \tag{D55}$$
$$= C_3 \mu_p^{\alpha_3}, \tag{D56}$$

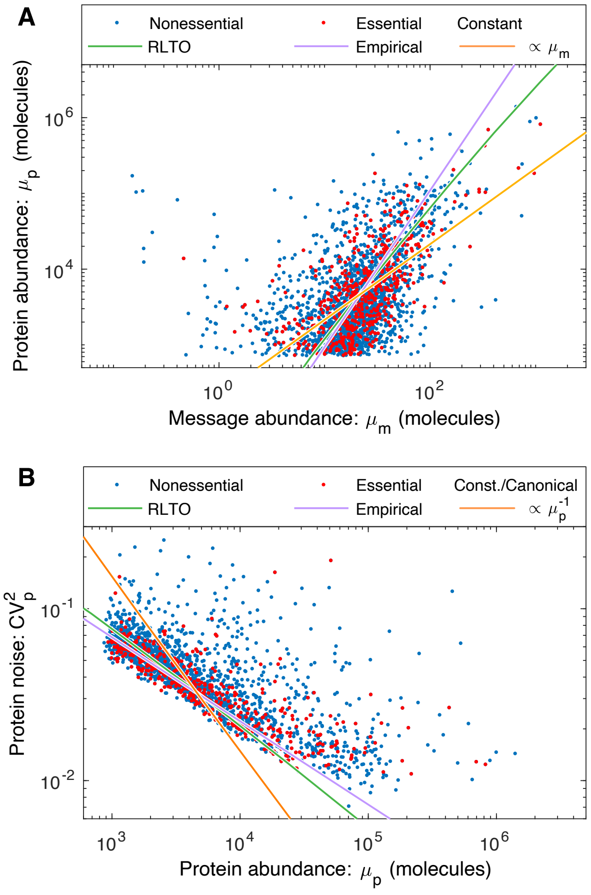

FIG. S14. **Panel A: Three competing models for protein abundance in yeast.** The empirical model (purple) fits the slope and the y offset. The RLTO (green) and constant-translation-efficiency (orange) models fit a parameter corresponding to the y offset only. As discussed in the analysis of the proteome fraction, the RLTO model qualitatively captures the scaling of the protein abundance with message number better than the constant translation efficiency model; however, the predicted fit does not correspond to the optimal power law, which is represented by the empirical model. The protein abundance has a cutoff near 10^1 due to autofluorescence [24]. **Panel B: Predictions of the noise-protein abundance relation.** Using each competing protein abundance model, the noise-protein abundance relation can be predicted using Eq. D14. The canonical noise model (Eq. D12) fails to capture even the scaling of the noise. In contrast, both the RLTO and empirical models quantitatively predict both the scaling and magnitude of the noise. The empirical model has the highest performance, presumably due to its two-parameter fit to the protein abundance in Panel A. A fit accounting for the noise floor is shown in Fig. S12.

where the last line defines two new parameters: a coefficient C_3 and an exponent α_3. The resulting parameters and uncertainties are:

$$\alpha_3 \equiv -1/\alpha_0, \quad \text{(D57)}$$
$$= -0.48 \pm 0.01, \quad \text{(D58)}$$
$$C_3 \equiv C_0^{1/\alpha_0} \ln 2, \quad \text{(D59)}$$
$$= 1.9 \pm 0.1, \quad \text{(D60)}$$

where the uncertainties are the estimated standard errors.

6. Results: Parameter-free prediction of noise-abundance in yeast

The fit of the competing protein-message abundance models are shown in Fig. 3A. Using each model, we can now predict the relation between protein abundance and noise without additional fitting parameters. The predictions of the three competing models are compared to the experimental data in Fig. 3B.

In both its ability to capture the protein abundance and predict the noise, the RLTO model vastly outperforms the constant-translation-efficiency model. The purely empirical model that best captures the protein abundance data, due to directly fitting both the y-offset and slope, also performs best in predicting the noise. It is important to emphasize that the prediction of the noise in all models is non-trivial since there are no free parameters fit, once the protein abundance relation is determined. We therefore conclude that the noise model (Eq. D14) quantitatively predicts the observed noise from the message number and that eukaryotic noise has non-canonical scaling due to load balancing.

7. Discussion: Implications of noise

What are the biological implications of gene expression noise? Many important proposals have been made, including bet-hedging strategies, the necessity of feedback in gene regulatory networks, *etc.* [54]. Our model suggests that robustness to noise fundamentally shapes the central dogma regulatory program. With respect to message number, the one-message-rule sets a lower bound on the transcription rate of essential genes. (See Fig. 4B.) With respect to protein expression, robustness to noise has two important implications: Protein overabundance significantly increases protein levels above what would be required in the absence of noise and therefore reshapes the metabolic budget. (See Fig. 4A.) Robustness to noise also gives rise to load balancing, the proportionality of the optimal transcription and translation rates. (See Fig. 4C.) Not only does robustness to noise affect central dogma regulation, but there is an important reciprocal effect: Load balancing changes the global scaling relation between noise and protein abundance. (See Fig. S14B.)

[1] H. G. S. Joseph W. Lengeler, Gerhart Drews, ed., *Biology of the Prokaryotes* (Georg Thieme Verlag, Rüdigerstrasse 14, D-70469 Stuttgart, Germany, 1998).
[2] D. Huang, T. Lo, H. Merrikh, P. A. Wiggins, *Phys Rev E* **105**, 014420 (2022).
[3] D. Huang, *et al.*, *Nat Commun* **14**, 1762 (2023).
[4] Y. Taniguchi, *et al.*, *Science* **329**, 533 (2010).
[5] R. Balakrishnan, *et al.*, *Science* **378**, eabk2066 (2022).
[6] M. Kafri, E. Metzl-Raz, G. Jona, N. Barkai, *Cell Rep* **14**, 22 (2016).
[7] E. Metzl-Raz, M. Kafri, G. Yaakov, N. Barkai, *G3 (Bethesda)* **10**, 3229 (2020).
[8] S. Ghaemmaghami, *et al.*, *Nature* **425**, 737 (2003).
[9] B. Schwanhäusser, *et al.*, *Nature* **473**, 337 (2011).
[10] J. Hausser, A. Mayo, L. Keren, U. Alon, *Nat Commun* **10**, 68 (2019).
[11] G. Arfken, *Mathematical Methods for Physicists* (Academic Press, Inc., San Diego, 1985), third edn.
[12] J. A. Bernstein, A. B. Khodursky, P.-H. Lin, S. Lin-Chao, S. N. Cohen, *Proc Natl Acad Sci U S A* **99**, 9697 (2002).
[13] H. Chen, K. Shiroguchi, H. Ge, X. S. Xie, *Mol Syst Biol* **11**, 808 (2015).
[14] A. Bartholomäus, *et al.*, *Philos Trans A Math Phys Eng Sci* **374** (2016).
[15] R. Milo, *Bioessays* **35**, 1050 (2013).
[16] B. Alberts, *et al.*, *Molecular Biology of the Cell* (Garland, 2002), fourth edn.
[17] L. L. Chia, C. McLaughlin, *Mol Gen Genet* **170**, 137 (1979).
[18] V. Pelechano, S. Chávez, J. E. Pérez-Ortín, *PLoS One* **5**, e15442 (2010).
[19] B. Futcher, G. I. Latter, P. Monardo, C. S. McLaughlin, J. I. Garrels, *Mol Cell Biol* **19**, 7357 (1999).
[20] G. M. Cooper, *The Cell: A Molecular Approach. 2nd edition* (Sinauer Associates 2000, 2000).
[21] E. Yang, *et al.*, *Genome Res* **13**, 1863 (2003).
[22] R. Milo, P. Jorgensen, U. Moran, G. Weber, M. Springer, *Nucleic Acids Res* **38**, D750 (2010).
[23] T. Baba, H.-C. Huan, K. Datsenko, B. L. Wanner, H. Mori, *Methods Mol Biol* **416**, 183 (2008).
[24] J. R. S. Newman, *et al.*, *Nature* **441**, 840 (2006).
[25] W. R. Blevins, *et al.*, *Sci Rep* **9**, 11005 (2019).
[26] L. M. Hereford, M. Rosbash, *Cell* **10**, 453 (1977).
[27] T. von der Haar, *BMC Syst Biol* **2**, 87 (2008).
[28] D. Zenklusen, D. R. Larson, R. H. Singer, *Nat Struct Mol Biol* **15**, 1263 (2008).
[29] F. Miura, *et al.*, *BMC Genomics* **9**, 574 (2008).
[30] J. van Leeuwen, *et al.*, *Mol Syst Biol* **16**, e9828 (2020).
[31] L. M. F. de Godoy, *et al.*, *Nature* **455**, 1251 (2008).
[32] M. Uhlén, *et al.*, *Science* **347**, 1260419 (2015).
[33] V. E. Velculescu, *et al.*, *Nat Genet* **23**, 387 (1999).
[34] T. Wang, *et al.*, *Science* **350**, 1096 (2015).
[35] S. Y. Gerdes, *et al.*, *J Bacteriol* **185**, 5673 (2003).
[36] E. C. A. Goodall, *et al.*, *mBio* **9** (2018).
[37] P. Mehta, S. Casjens, S. Krishnaswamy, *BMC Microbiol* **4**, 4 (2004).
[38] F. Crick, *Nature* **227**, 561 (1970).
[39] J. L. Hargrove, F. H. Schmidt, *FASEB J* **3**, 2360 (1989).
[40] A. L. Koch, H. R. Levy, *J Biol Chem* **217**, 947 (1955).
[41] M. Martin-Perez, J. Villén, *Cell Syst* **5**, 283 (2017).
[42] D. Gillespie, *Physica A* **188**, 404 (1992).
[43] D. T. Gillespie, *The Journal of Physical Chemistry* **81**, 2340 (1977).
[44] J. Paulsson, M. Ehrenberg, *Phys Rev Lett* **84**, 5447 (2000).
[45] N. Friedman, L. Cai, X. S. Xie, *Phys Rev Lett* **97**, 168302 (2006).
[46] (2023).
[47] A. Bar-Even, *et al.*, *Nat Genet* **38**, 636 (2006).
[48] M. B. Elowitz, A. J. Levine, E. D. Siggia, P. S. Swain, *Science* **297**, 1183 (2002).
[49] P. S. Swain, M. B. Elowitz, E. D. Siggia, *Proc Natl Acad Sci U S A* **99**, 12795 (2002).
[50] C. R. Rao, *Bulletin of Calcutta Mathematical Society* **37**, 81 (1945).
[51] H. Cramer, *Mathematical methods of statistics.* (Princeton University Press., 1946).
[52] P.-J. Lahtvee, *et al.*, *Cell Syst* **4**, 495 (2017).
[53] K. H. Hellton, M. Thoresen, *Scandinavian Journal of Statistics* **41**, 1051 (2014).
[54] J. M. Raser, E. K. O'Shea, *Science* **309**, 2010 (2005).
